# NINJ1 blocks HSV-1 entry into macrophages to impact viral replication and immunity

**DOI:** 10.1038/s44319-025-00638-8

**Published:** 2025-11-19

**Authors:** Ella Hartenian, Magalie Agustoni, Petr Broz

**Affiliations:** Department of Immunobiology, University of Lausanne, Lausanne, 1066 Switzerland

**Keywords:** HSV-1, NINJ1, Cell Death, Virology, Restriction Factors, Immunology, Microbiology, Virology & Host Pathogen Interaction

## Abstract

Restriction factors block multiple stages of viral infection. Here we describe how Ninjurin1 (NINJ1) controls HSV-1 infection of macrophages, a key cell type that protects mice against infection. We observe that *Ninj1*^–/–^ mouse macrophages are more susceptible to HSV-1 infection than WT cells. Given the role of NINJ1 during cell death, we investigate whether its antiviral activity is linked to this function. Surprisingly, we do not observe differences in cell death at early timepoints post HSV-1 infection between genotypes. Instead, we attribute the higher infection rate of *Ninj1*^–/–^ macrophages to enhanced entry, with more viral particles entering each cell and a greater fraction of infected cells. The increased viral loads in *Ninj1*^–/–^ cells result in higher ISG and cytokine RNA expression, which we ascribe to both TLR signaling and STING-mediated recognition. Cytokine secretion, however, is severely dampened in infected *Ninj1*^–/–^ cells, pointing to greater viral replication suppressing the induction of inflammation. In conclusion, NINJ1 acts as a gatekeeper for HSV-1 entry in macrophages, impacting the inflammatory phenotype associated with HSV-1 infection.

## Synopsis

NINJ1 blocks HSV-1 entry into mouse macrophages independent of its role in cell death, resulting in greater secretion of pro inflammatory cytokines downstream of infection.

- Mouse macrophages lacking NINJ1 are more permissive to HSV-1 entry, with higher viral gene expression and virion production.
- NINJ1 controls HSV-1 infection independently of its role in cell death.
- Greater levels of HSV-1 replication in NINJ1-deficient cells leads to the release of fewer pro inflammatory cytokines.

## Abstract (web summary)

NINJ1 blocks HSV-1 entry into mouse macrophages independent of its role in cell death, resulting in greater expression of pro inflammatory cytokines downstream of infection.

## Graphical abstract

**Figure:**
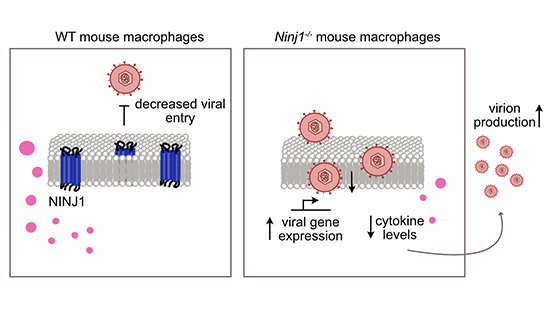

**Figure:**
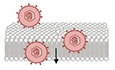

## Introduction

Herpes simplex virus 1 (HSV-1) affects four in five people worldwide, making it one of the most ubiquitous viruses. Infections are lifelong, and symptoms vary between individuals, from asymptomatic to blisters, to severe outcomes, including blindness and encephalitis. HSV-1 initially infects keratinocytes and fibroblasts, then gains access to axons of the peripheral nervous system, where it establishes latency in sensory neurons and undergoes rounds of reactivation. When infection spreads beyond sensory neurons to the central nervous system (CNS), patients experience encephalitis—brain swelling and potentially debilitating symptoms.

Macrophages are innate immune sentinels and express many interferon-stimulated genes (ISGs), pattern recognition receptors (PRRs) and cell death pathways components. While keratinocytes, fibroblasts and neurons are relevant cell types for HSV-1 infections, experiments in mice, especially in the brain, have consistently also shown a role for monocytic cells. Indeed, a recent single-cell analysis of an encephalitis model found that monocyte-like cells represented more than half of infected cells in the brain (Ding et al, 2025). Monocyte-like cells began infiltrating the brain 4 days post infection and peaked at 7 days post infection. A second study found that depleting macrophages from mice during a systemic intravenous infection or a vaginal infection resulted in decreased early control of the virus in the systemic case and death of the animals in both models (Lang et al, 2020). Furthermore, depleting microglia from mouse brains renders animals more susceptible to HSV-infection due to a dampened interferon (IFN) response (Katzilieris-Petras et al, 2022; Reinert et al, 2016). HSV-1 infection of human monocyte-derived macrophages also demonstrated cytokine production after infection (Melchjorsen et al, 2006). Together, these studies highlight the important role monocytic cells play in determining the outcome of infection.

One mechanism that host cells employ to limit viral replication is the induction of programmed cell death pathways, including pyroptosis, apoptosis and necroptosis. These pathways can act as sensors for infection. Suppression of apoptosis by viruses, on the other hand, engages necroptosis, which serves as a backup pathway of cell death in case apoptosis is inhibited (Kaiser et al, 2013). Previous reports have shown that HSV-1 modulates multiple cell death pathways during infection, including necroptosis, apoptosis and pyroptosis, inhibiting caspase-8, AIM2 and NLRP1-driven detection (Guo et al, 2018, 2022; Maruzuru et al, 2018; Parameswaran et al, 2024; Aubert and Blaho, 2001). Immune cells are also susceptible to cell death during infection. During a CNS model of mouse HSV-1 infection, microglia were shown to die by apoptosis, which had a beneficial effect on viral replication as it reduced the production of type-I IFN by these cells (Reinert et al, 2020).

Here, we describe a role for Ninjurin-1 (NINJ1), a plasma membrane resident protein highly expressed in the monocyte lineage of both human and mouse, in the restriction of HSV-1 infection. NINJ1 was originally described to be important for cell-to-cell adhesion (Lee et al, 2010), but has recently been shown to be essential for plasma membrane rupture and cellular lysis, the terminal event of multiple cell death pathways including apoptosis, ferroptosis, pyroptosis and necroptosis (Kayagaki et al, 2021; Ramos et al, 2024). NINJ1 undergoes self-oligomerization during cell death to form membrane-embedded filaments with a distinct hydrophilic and hydrophobic face. This feature allows NINJ1 filaments to create large pore-like lesions in the plasma membrane that release large cellular molecules such as danger-associated molecular patterns (DAMPs), chemokines, cytokines and nucleic acids (Kayagaki et al, 2021; Zhu et al, 2025). Most recently, NINJ1 has been shown to be a sensor of mechanical membrane stress, such that plasma membrane NINJ1 acts as “weak links” that break upon additional stress (Zhu et al, 2025), such as during necrosis-associated cell swelling (preprint:Hartenian et al, 2024). NINJ1 has further been shown to be important for the release of norovirus, a non-enveloped virus that both triggers and requires cell death for egress (Wang et al, 2023).

Here, we report that *Ninj1*-deficiency in mouse macrophages leads to enhanced infection with HSV-1. By monitoring HSV-1 GFP infection dynamics, we see earlier and stronger infection of *Ninj1*^*–/–*^macrophages as compared to WT controls. Although we observe initial differences in replication shortly after infection, *Ninj1*-deficiency has consequences for all stages of the HSV-1 lifecycle and eventually results in higher production of infectious viral particles. To understand how NINJ1 restricts HSV-1 infections, we first investigate if this is related to the role of NINJ1 in cell death. However, we observe that infected *Ninj1*-deficient cells do not die more or earlier than WT controls at early timepoints in infection, at which we already observe a difference in infection rates. Further exploring the effects of *Ninj1*-deficiency, we find that HSV-1 entry of *Ninj1*^*–/–*^ macrophages is enhanced, with a higher infection rate and more viral particles entering each cell on average. Finally, we determine the consequences of the altered entry, measuring higher ISG RNA expression in infected *Ninj1*^–/–^ cells, which we ascribe to both TLR signaling and STING-mediated recognition of nucleic acids. Cytokine protein production, on the other hand, is decreased in *Ninj1*^–/–^ cells, which we attribute to the consequences of higher viral replication and thus higher levels of suppression. Together, this indicates that during infection of mouse macrophages, NINJ1 reduces HSV-1 entry, which alters the inflammatory potential of macrophages.

## Results and discussion

### NINJ1 controls the susceptibility of macrophages to HSV-1

**Figure 1:**
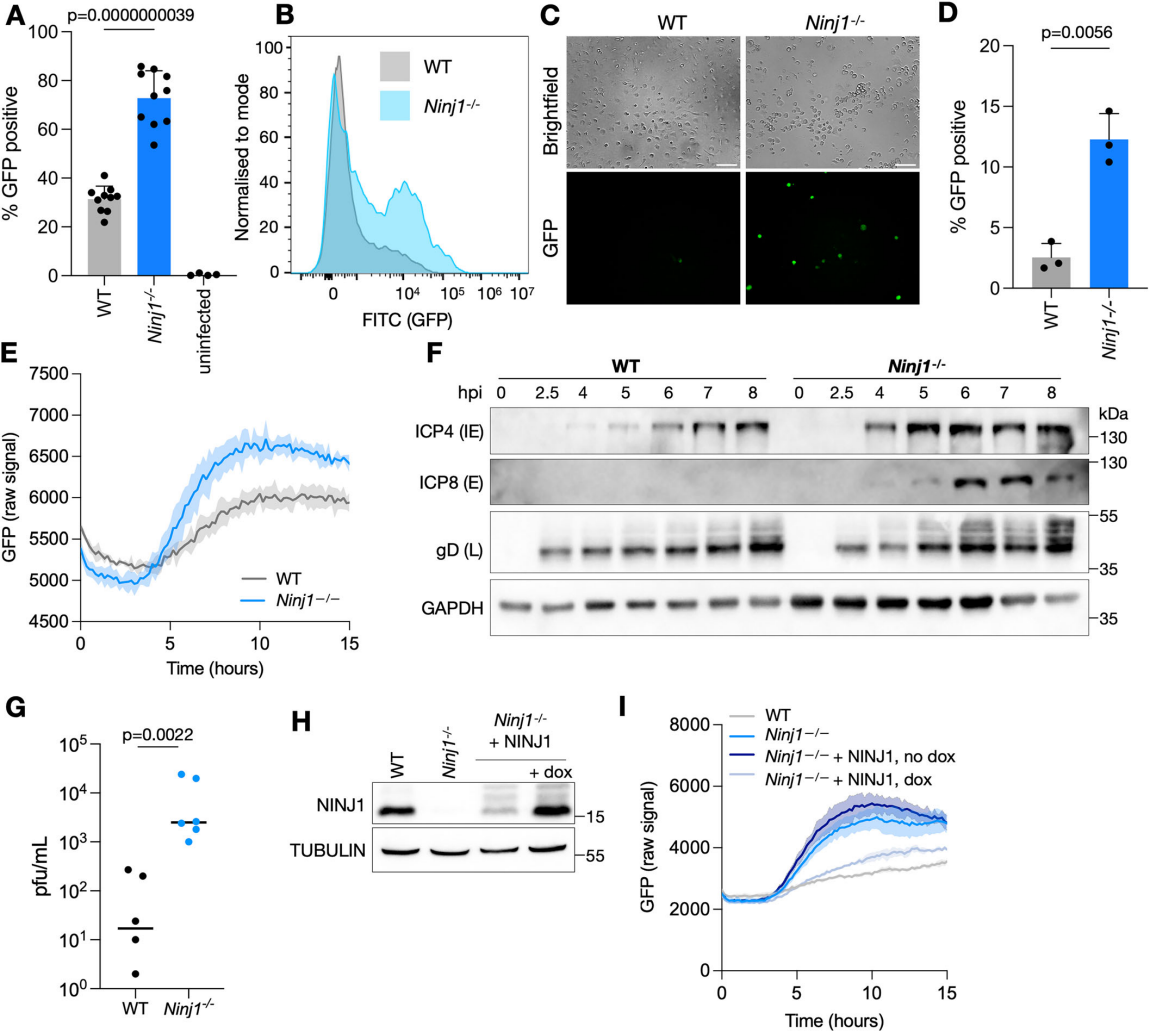
NINJ1 controls the susceptibility of mouse macrophages to HSV-1. (**A**) WT and *Ninj1*^*–/–*^ iBMDMs were infected with HSV-1 GFP for 6 h at a MOI of 10 and subjected to flow cytometry to determine the percentage of infected cells. (**B**) WT and *Ninj1*^*–/–*^ iBMDMs were infected with HSV-1 GFP for 6 h at an MOI of 10 and subjected to flow cytometry, with the percentage of GFP-positive cells shown. (**C**) WT and *Ninj1*^*–/–*^ primary BMDMs were infected and visualized by microscopy after 6 h. Representative data from one experiment is shown (*n* = 5). (**D**) WT and *Ninj1*^*–/–*^ primary BMDMs were infected and subjected to flow cytometry with the percentage of GFP-positive cells shown. (**E**) WT and *Ninj1*^*–/–*^ iBMDMs were infected at a MOI of 10, and the GFP signal was measured every 10 m with a Citation5 Plate Reader. (**F**) WT and *Ninj1*^*–/–*^ iBMDMs were infected with HSV-1 GFP for the indicated timepoints, then subjected to immunoblotting for the indicated proteins. (**G**) Plaque assays from supernatant of WT and *Ninj1*^*–/–*^ iBMDMs infected with HSV-1 GFP for 24 h. (**H**) Immunoblot analysis of NINJ1 levels in WT and *Ninj1*^*–/–*^ iBMDMs complemented with a doxycycline (dox) inducible NINJ1 in the presence and absence of dox. TUBULIN serves as a loading control. (**I**) HSV-1 GFP signal measured every 10 m in WT, *Ninj1*^*–/–*^ iBMDMs and *Ninj1*^*–/–*^ iBMDMs complemented with a dox inducible NINJ1 ± dox with a Citation5 Plate Reader. Data information: In (**A**, **D**, **E**) data were presented as mean ± SD. Individual data points show biological replicates. A: unpaired *t*-test, *n* = 10, B: 1 representative experiment from 10 is shown, (**C**) one representative biological replicates of 3 is shown, (**D**) *n* = 3, unpaired *t*-test, (**E**) mean ± SD from 3 technical replicates is plotted, experiment is representative of four biological replicates, (**F**) 1 representative experiment of three biological replicates is shown, (**G**) dots are 1–2 technical replicates from three biological replicates with a line at the mean, Kolmogorov–Smirnov test, (**H**) one representative experiment of two biological replicates is shown, (**G**) mean ± SD from three technical replicates is plotted, experiment is representative of three biological replicates. Source data are available online for this figure.

NINJ1 is a membrane protein that functions in cell adhesion and cell death. As both processes are important for pathogens to successfully invade and replicate in host cells, we sought to determine if NINJ1 plays a role during infection with HSV-1. We therefore infected immortalized bone marrow-derived macrophages (iBMDMs) from WT mice or mice deficient in *Ninj1*^*–/–*^ with HSV-1 expressing a green fluorescence protein (HSV-1 GFP) under the control of the immediate early protein promoter ICP47 (see Reagents and Tools Table). Measuring the percentage of GFP-positive (GFP+) cells as a readout for infection, we found that *Ninj1*-deficiency resulted in twice as many infected cells compared to WT cells, as measured by flow cytometry at 6 h post infection (hpi) (Fig. 1A). Further analysis of infected cells showed that in the absence of NINJ1, more cells were infected and they also trended towards a higher mean fluorescent intensity (MFI), indicating that NINJ1 expression either resulted in dampened GFP expression or altered early stages of infection (Figs. 1B and EV1A). To ensure that this effect was not an artifact due to the immortalization of the macrophages, we next examined infection of primary bone marrow-derived mouse macrophages (BMDMs) derived from WT or *Ninj1*^*–/–*^ mice. Widefield microscopy showed an increase in GFP+ cells in the absence of NINJ1 upon infection with HSV-1-GFP (Fig. 1C), and quantification by flow cytometry confirmed a threefold increase in the fraction of GFP+ *Ninj1*^*–/–*^ cells as compared to WT (Fig. 1D), consistent with the results from immortalized BMDMs. To understand the kinetics of HSV-1 control by NINJ1, we analyzed the GFP signal intensity of infected cells every 10 m from the time of infection until 15 hpi. We observed that after an initial drop in the GFP signal, which occurred in both genotypes, there was a stronger increase in GFP intensity in *Ninj1*^*–/–*^ cells as compared to the WT cells, starting around 4 hpi and plateauing around 11 hpi (Fig. 1E), consistent with our other measurements of HSV-1 GFP signal.

**Figure EV1:**

NINJ1 controls the susceptibility of mouse macrophages to HSV-1. (**A**) WT and *Ninj1*^*–/–*^ iBMDMs were infected for 6 h with HSV-1 GFP, subjected to flow cytometry and the median fluorescence intensity was measured in FlowJo. (**B**) *Ninj1*^*–/–*^ iBMDMs ± integration of a blasticidin resistance transgene were infected with HSV-1 GFP, and GFP signal was measured every 10 min on a Citation5 Plate Reader. Data information: (**A**) dots are biological replicates with a line connecting data collected as part of the same experiment, (**B**) line is mean ± SD from three technical replicates, representative of two biological replicates.

To confirm that the increase in HSV-1 GFP signal corresponded to an increase in viral gene products, we analyzed the expression of three viral proteins— ICP4 (immediate early), ICP8 (early), and gD (leaky late)—from 2.5 hpi to 8 hpi by immunoblotting in infected iBMDMs. Consistent with a stronger GFP signal of HSV-1 in the absence of NINJ1, *Ninj1*^*–/–*^ macrophages not only showed higher expression levels of all three proteins, but also earlier expression of ICP4 and ICP8 (Fig. 1F). We next queried if the higher levels of viral protein expression resulted in the production of more infectious virions. We therefore collected supernatants from cells infected for 24 h and measured the number of infectious virions by plaque assay. We observed two logs higher titer from *Ninj1*^*–/–*^ cells as compared to WT cells (Fig. 1G), consistent with a higher infection rate of *Ninj1*^*–/–*^ cells.

To confirm that the observed phenotype was due to *Ninj1*-deficiency alone and not an off-target effect, we transduced *Ninj1*^*–/–*^ iBMDMs with a vector expressing *Ninj1* under a doxycycline(dox)-inducible promoter. Treatment with dox induced expression of NINJ1 to levels comparable to WT cells (Fig. 1H). We next infected WT, *Ninj1*^*–/–*^ and *Ninj1*^*–/–*^ + *Ninj1* iBMDMs, in the presence or absence of dox, with HSV-1 GFP and measured the GFP signal over time. Complementing *Ninj1*-deficiency reduced the GFP signal to levels comparable to WT cells, confirming that *Ninj1*-deficiency was indeed the reason for increased viral replication (Fig. 1I). In the complemented cells in the absence of dox, the GFP signal followed the same trend as in *Ninj1*^*–/–*^ cells, but was slightly attenuated, likely due to leaky expression of the construct (Fig. 1H). We also checked if overexpression of another, unrelated protein, could alter HSV-1 replication. Therefore, we lentivirally integrated a blasticidin resistance cassette into *Ninj1*^*–/–*^ cells. After blasticidin selection, infection with HSV-1 GFP in the presence and absence of the blasticidin transgene resulted in similar levels of GFP expression (Fig. EV1B), demonstrating that overexpression of an unrelated protein does not alter HSV-1 replication. In summary, the results show that NINJ1 controls the susceptibility of mouse BMDMs to HSV-1.

### NINJ1 prevents infection in a cell-death-independent manner

**Figure 2:**
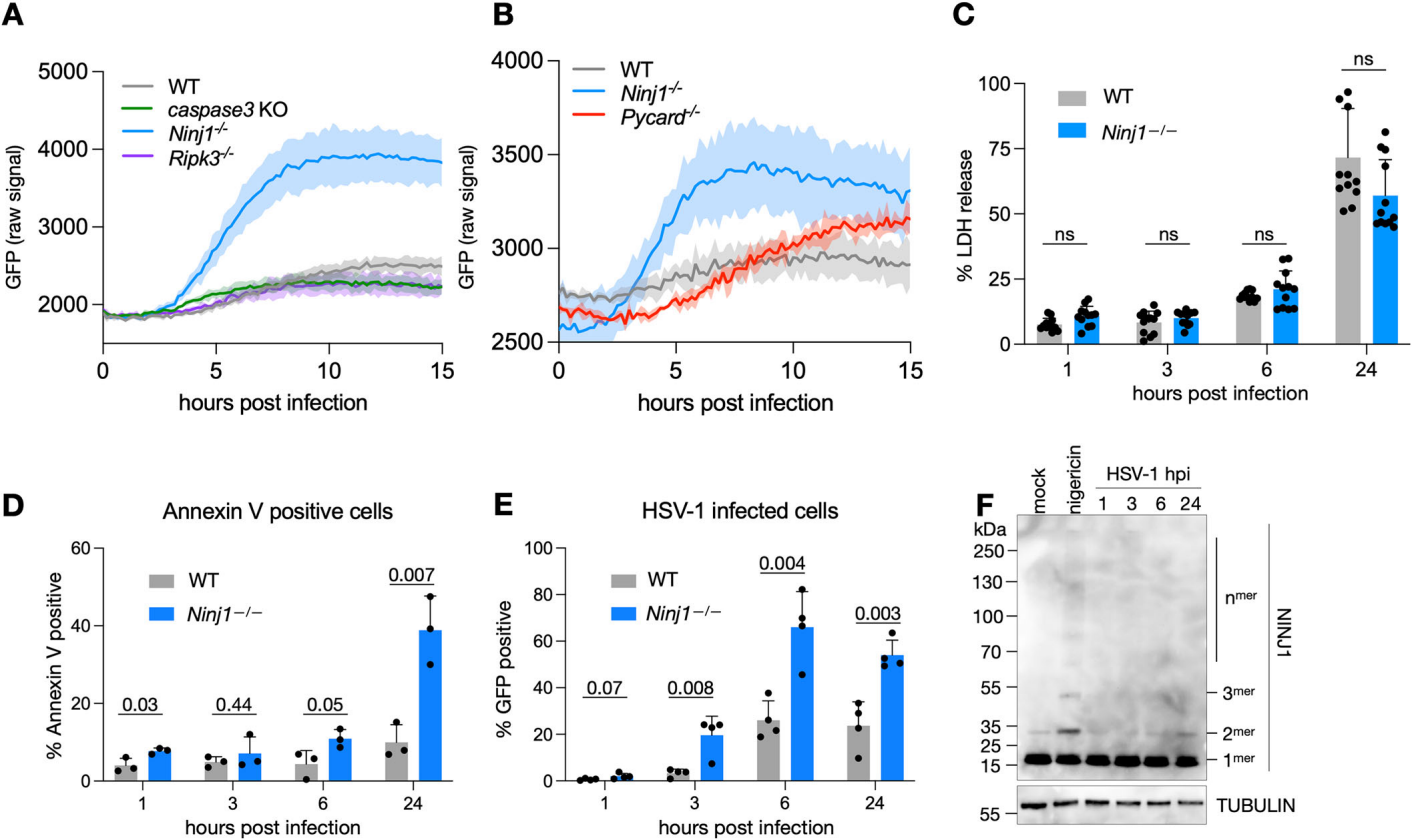
NINJ1 prevents infection in a cell-death-independent manner. (**A**, **B**) The indicated genotypes of iBMDMs were infected with HSV-1 GFP and GFP signal was monitored every 10 m on a Citation5 plate Reader. (**C**) Lactate dehydrogenase (LDH) release was measured from the supernatant of HSV-1 GFP-infected WT and *Ninj1*^*–/–*^ iBMDMs at the indicated hours post infection. (**D**, **E**) WT and *Ninj1*^*–/–*^ iBMDMs infected with HSV-1 GFP were stained with Annexin V and subjected to flow cytometry, analyzing the Annexin V signal (**D**) and the percentage of infected cells (**E**). (**F**) Crosslinking immunoblot of WT primary BMDMs infected with HSV-1 GFP for the indicated periods of time. H3 serves as a loading control, and nigericin shows oligomerized NINJ1 upon cell death as a positive control. Data information: In (**A**, **B**) mean ± SD from three technical replicates is plotted, experiments are representative of three biological replicates. In (**C**–**E**), bars show the mean ± standard deviation. (**C**) Plotted dots are technical replicates from four biological replicates, unpaired *t*-test, ns not significant *n* = 12, (**D**, **E**) unpaired *t*-test *n* = 3–4, (**F**) one representative experiment of three biological replicates is shown. Source data are available online for this figure.

Given the key role of NINJ1 in cell lysis downstream of multiple programmed cell death (PCD) pathways and the implication of several of these pathways in the defense against HSV-1, we wished to determine if the inactivation of any PCD pathway phenocopied *Ninj1*-deficiency, which would implicate the respective pathway as a driver of NINJ1 activation. We therefore tested iBMDMs deficient for ASC (encoded by the gene *Pycard*), a key inflammasome adaptor protein needed for inflammasome-induced pyroptosis; RIPK3 (receptor interacting protein kinase 3), a critical necroptosis adaptor protein, and are unable to undergo necroptosis; and caspase-3, the apoptotic executioner caspase. We infected these cells in parallel with WT and *Ninj1*^*–/–*^ iBMDMs with HSV-1 GFP and measured the GFP signal over time. While we observed a clear increase in the GFP signal in the *Ninj1*^*–/–*^ cells, we did not detect a comparable increase in any of the other knock-out cell lines, suggesting that the individual removal of each of these cell death pathways did not phenocopy the *Ninj1*^*–/–*^ cells (Fig. 2A,B).

We then asked if the absence of NINJ1 delayed macrophage death, allowing HSV-1 to replicate to higher levels. We therefore measured the release of lactate dehydrogenase (LDH), a marker for lytic cell death and NINJ1 activation, from HSV-1-infected WT and *Ninj1*^*–/–*^ cells at 1, 3, 6, and 24 hpi. Even though *Ninj1*-deficiency had an impact on viral replication as early as 5–6 hpi (Fig. 1), we did not observe any difference in LDH release between WT and *Ninj1*^*–/–*^ macrophages at these timepoints (Fig. 2C). By 24 hpi, LDH release had increased to ~60% in both genotypes, with no significant differences between them.

Cell lysis is the terminal stage of lytic cell death pathways such as pyroptosis and necroptosis. Apoptosis, by contrast, does not induce cell lysis, and thus it is possible that WT and *Ninj1*^*–/–*^ cells display different levels of apoptosis. We thus used Annexin-V staining, which is commonly used to detect the exposure of phosphatidyl serine (PS) on the plasma membrane. Annexin V staining showed low levels of PS exposure in both HSV-1-infected WT and *Ninj1*^*–/–*^ cells at 1–6 hpi (Fig. 2d). At 24 hpi, the Annexin V signal increased significantly in the *Ninj1*^*–/–*^ cells while remaining lower in the WT cells. The increase in the *Ninj1*^*–/–*^ cells corresponded to a higher infection rate, which peaked in the *Ninj1*^*–/–*^ cells at 6 h and declined slightly by 24 h (Fig. 2E).

A hallmark of NINJ1 activation during cell death is the oligomerization of the protein into filaments, which are required to induce cell lysis. Since our data suggest that NINJ1 plays a cell-lysis independent role during HSV-1 infections, we wished to confirm if NINJ1 oligomerized during HSV-1 infection by detecting oligomeric NINJ1 by crosslinking immunoblots during infection. As a control, we included cells treated with nigericin, which induces pyroptotic cell death. Consistent with published reports (Kayagaki et al, 2021; Degen et al, 2023), we found that nigericin treatment resulted in the formation of NINJ1 dimers, trimers and higher-order oligomers, indicating NINJ1 activation (Fig. 2F). By contrast, we didn’t observe any higher-order oligomers in HSV-1 infected cells. A low level of NINJ1 dimers was detectable, but this was comparable to untreated controls (Fig. 2F). This indicates that infection of WT macrophages with HSV-1 does not lead to oligomerization between 1 and 24 hpi, further confirming that NINJ1 must control HSV-1 infection independently of its role as an executioner of lytic cell death.

### Early stages of the viral lifecycle are impacted by the loss of NINJ1

**Figure 3:**
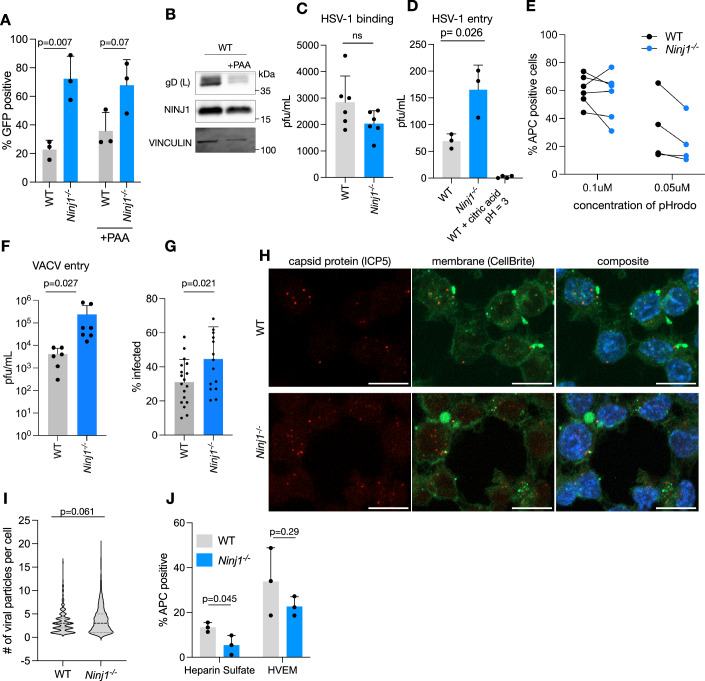
Viral entry is enhanced in the absence of NINJ1. (**A**) WT and *Ninj1*^*–/–*^ iBMDMs were infected for 18 h with HSV-1 GFP in the presence or absence of phosphonoacetic acid (PAA) and subjected to flow cytometry to measure the fraction of GFP+ cells. (**B**) Western blot of the leaky late protein gD, NINJ1, and VINCULIN in the presence and absence of PAA at 18 hpi. Representative of two biological replicates. (**C**) Plaque assay of HSV-1 GFP incubated with WT and *Ninj1*^*–/–*^ iBMDMs for 1 h at 4 °C to measure viral binding. (**D**) Plaque assay of HSV-1 GFP incubated with WT and *Ninj1*^*–/–*^ iBMDMs for 15 m and then washed with low-acid citric acid buffer to measure entry. Citric acid condition was HSV-1 and citric acid buffer added at the same time. (**E**) WT and *Ninj1*^*–/–*^ iBMDMs were subjected to flow cytometry after incubation with the indicated concentration of fluorescent dextrans for 30 m to monitor endocytosis. (**F**) WT and *Ninj1*^*–/–*^ iBMDMs were infected with VACV for 10 m, washed, and intracellular virions were plaqued. (**G**–**I**) Confocal fluorescent microscopy of cells infected with HSV-1 GFP for 45 m and stained with ICP5, cell brite and Hoechst. (**G**) Percentage of cells infected. (**H**) Representative images, scale bar 10 um. (**I**) Plot of the number of capsids (ICP5 positive foci) per WT and *Ninj1*^*–/–*^ cell, defined using cellbrite stain. (**J**) WT and *Ninj1*^*–/–*^ iBMDMs were subjected to flow cytometry after being incubated with Heparin Sulfate and HVEM primary antibodies. Data information: (**A**, **D**, **F**, **J**) mean ± SD are plotted with individual dots showing biological replicates, while (**C**) shows two technical replicates from three biological replicates. (**A**, **D**, **F**, **G**, **J**) unpaired *t*-test. (**B**) one biological replicate representative of 2, (**E**) dots show biological replicates with lines indicating those performed together, (**G**) each dot represents one of 14–17 fields of view taken from three biological replicates for a total of 750–950 cells, (**I**) violin plot with a solid line at the mean and dotted lines at quartiles, Kolmogorov–Smirnov test. Source data are available online for this figure.

Having ruled out an antiviral effect of NINJ1 related to its role in cell death, we next wished to distinguish at what stage of the viral lifecycle NINJ1 exerts its antiviral effect. Since we had observed a difference in GFP expression as early as 6 hpi (Fig. 1A,C,D) and a difference in ICP8 expression at 4 hpi (Fig. 1E), we speculated that NINJ1 modulated early events in the viral lifecycle. However, to rule out that *Ninj1*-deficiency affected post-viral gene replication events, we treated WT and *Ninj1*^*–/–*^ cells with the viral DNA replication inhibitor Phosphonoacetic acid (PAA) during HSV-1 GFP infection and measured the GFP signal. PAA did not alter the higher infection rate of *Ninj1*^*–/–*^ cells at 18 hpi (Fig. 3A). Western blotting for viral gene products at 18 hpi showed reduced expression of the leaky late protein gD in the presence of PAA (Fig. 3B), confirming the efficacy of PAA. Together, these results indicate that NINJ1 restricts the HSV-1 lifecycle at a stage prior to viral DNA replication.

### NINJ1 blocks viral entry into cells

Having determined that NINJ1 acts upstream of viral DNA replication, we examined the early steps of viral infection, starting with binding, the initial step of viral entry. To measure the number of viral particles bound to WT and *Ninj1*^*–/–*^ cells, we incubated viral inoculum with cells at 4 °C, which allows viral binding but prevents entry, for 1 h, washed away unbound viral particles and compared the number of viral particles bound to cells by plaque assay. We observed slightly lower pfu/mL associated with *Ninj1*^*–/–*^ cells as compared to WT (Fig. 3C), although the difference was not significant.

We next examined viral entry. HSV-1 can enter cells both by direct membrane fusion and by endocytosis, which is then followed by the fusion of the viral particle with the endocytic membrane. Endocytic entry has been proposed to be the major pathway for entry into macrophages (Lang et al, 2020). Right after endocytic uptake, there is a short window of time in which viral particles remain intact and infectious within endocytic vesicles. We therefore speculated that if NINJ1 restricts viral entry, more infectious virions could be recovered from endosomes in the absence of NINJ1. To assess the level of entry into cells, we thus infected WT and *Ninj1*^*–/–*^ cells for 15 min (m) with HSV-1, treated cells with citric acid to inactivate all viral particles that had not entered cells and then determined the number of virions within endosomes by plaque assay. We observed three times as many viral particles in *Ninj1*^*–/–*^ cells as compared to WT cells (Fig. 3D), indicating that NINJ1 restricts HSV-1 entry into cells. To understand if this is a specific or general effect, we tested if *Ninj1-*deficiency impacted the endocytosis of fluorescent dextrans. We observed no change or even a slight decrease in endocytosis of *Ninj1*^*–/–*^ macrophages (Fig. 3E). We then asked if entry of Vaccina virus (VACV), another enveloped virus that can enter by endocytosis was altered in the absence of NINJ1. We therefore infected WT and *Ninj1*^*–/–*^ cells for 10 m with VACV and plaqued intact viral particles that had entered cells. Similar to HSV-1, we observed enhanced levels of VACV entry in *Ninj1*^*–/–*^ cells compared to WT controls (Fig. 3F), suggesting that NINJ1 may have a general antiviral role by restricting viral entry through endocytic compartments.

We next used confocal microscopy to quantify the fraction of cells positive for ICP5, a HSV-1 capsid protein, at 45 m post infection. This method detects virions that enter by both direct membrane fusion and endocytosis. Compared to WT cells, more *Ninj1*^*–/–*^ cells were found to be infected with HSV-1 (Fig. 3G,H). Moreover, counting the number of capsids inside each infected cell by microscopy showed that *Ninj1*^*–/–*^ cells also tended to harbor more capsids per cell than WT cells (Fig. 3I). This confirms that HSV-1 can both enter more cells in the absence of NINJ1 and that the number of successful entry events per cell increases.

Finally, we asked if the absence of NINJ1 alters the expression of known HSV-1 entry mediators. We compared the presence of HVEM, Heparin Sulfate and Nectin-1 on the surface of WT and *Ninj1*^*–/–*^ cells. There was slightly less Heparin Sulfate and HVEM detectable on the cell surface in *Ninj1*^*–/–*^ cells as compared to WT cells, but this difference was too small to explain higher viral entry into these cells (Fig. 3J). Nectin-1 levels were not detectable on either genotype, with two different antibodies. In summary, these data demonstrate that NINJ1 restricts the entry of HSV-1 into macrophages, and this does not depend on altered expression of known HSV-1 entry mediators.

### NINJ1 is highly expressed in primary macrophages and in primary mouse tissues

**Figure 4:**
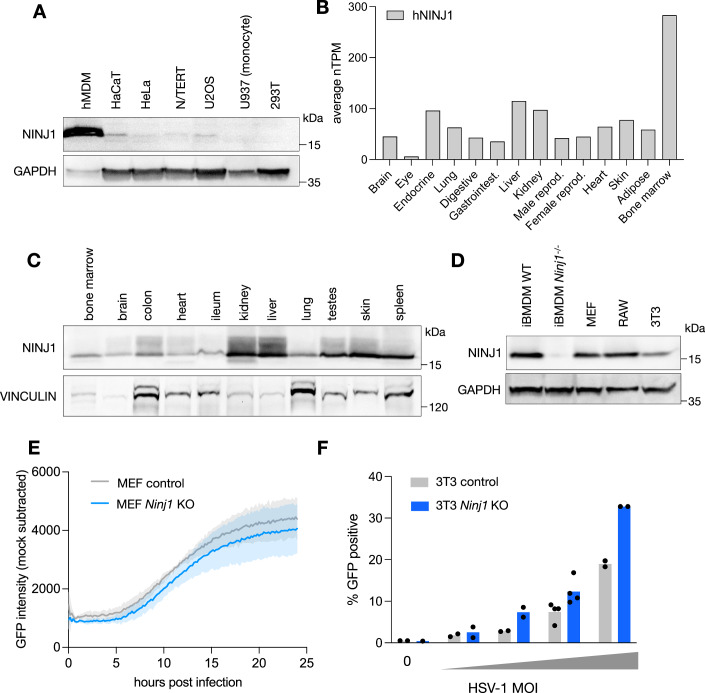
NINJ1 is highly expressed in primary macrophages and mouse tissue. (**A**) The indicated cell lines were subjected to immunoblot for human NINJ1 and GAPDH as a control. (**B**) NINJ1 transcripts per million across different human tissues. Data from the Human Protein Atlas. (**C**) Organ homogenates from a WT mouse were blotted for NINJ1 and VINCULIN as a loading control. (**D**) The indicated cell lines were subjected to an immunoblot for mouse NINJ1 and GAPDH as a control. (**E**) Infection of control (sgluciferase) and *Ninj1* KO MEFs with HSV-1 GFP was measured by detecting the GFP signal every 10 m on a Citation5 Plate Reader. (**F**) Infection rate of control (sgluciferase) and *Ninj1* KO 3T3s with increasing amounts of HSV-1 GFP for 8 h as measured by flow cytometry. Data information: (**A**, **D**) are representative of three biological replicates. (**C**) is representative of two mice, see Fig. EV2 for additional data. (**E**) Line is mean ± SD from three technical replicates, representative of three biological replicates, (**F**) bar is the mean with dots indicating biological replicates *n* = 2–4. Source data are available online for this figure.

**Figure EV2:**
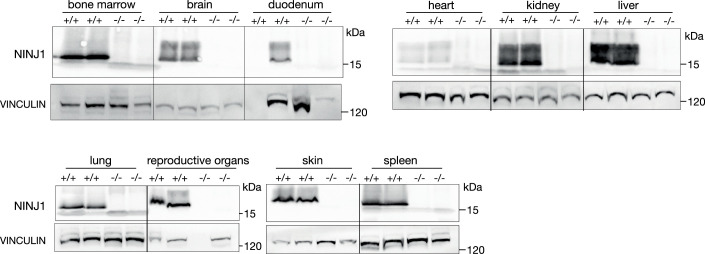
NINJ1 is highly expressed in primary macrophages and mouse tissue. Organ homogenates from two WT mice (+/+) and two *Ninj1*^*–/–*^ mice (−/−), one male (left) and one female (right) of each genotype, were subjected to immunoblot for NINJ1 expression across the indicated tissues.

Having characterized the role of NINJ1 as a gatekeeper for HSV-1 entry in macrophages, we wondered if it exerts the same role in other cell types. We thus examined NINJ1 expression across a panel of human and mouse cell lines and mouse tissue. We observed that the expression of NINJ1 in immortalized human cells lines was very low when compared to primary human monocyte-derived macrophages which had strong NINJ1 expression, consistent with previous reports (Borges et al, 2022) (Fig. 4A). Expression data from the human protein atlas also indicates that NINJ1 is most highly expressed in bone marrow lineage cells (Fig. 4B). In contrast, we detected NINJ1 expression across 11 different primary mouse tissues (Figs. 4C and EV2), indicating that NINJ1 is broadly expressed in mice. We also examined several mouse cell lines and found that NINJ1 was expressed in mouse embryonic fibroblasts (MEFs), RAW macrophages and 3T3 fibroblasts (Fig. 4D). To assess whether NINJ1 modulates HSV-1 infection in these cell types, as observed in macrophages, we infected CRISPR-generated NINJ1 knockout (KO) cells or KO controls with HSV-1. In MEFs, infection levels were comparable between NINJ1 KO and controls (Fig. 4E). In contrast, we observed elevated infection rates in NINJ1 KO 3T3s compared to control cells (Fig. 4F), suggesting a role for NINJ1 in infection of these cells. In summary, these results demonstrate that mouse NINJ1 is widely expressed in both primary tissues and various cell lines, including RAW macrophages and fibroblasts, and that its impact on HSV-1 infection is cell-type-specific.

### HSV-1-induced cytokine secretion is suppressed in cells lacking NINJ1

**Figure 5:**
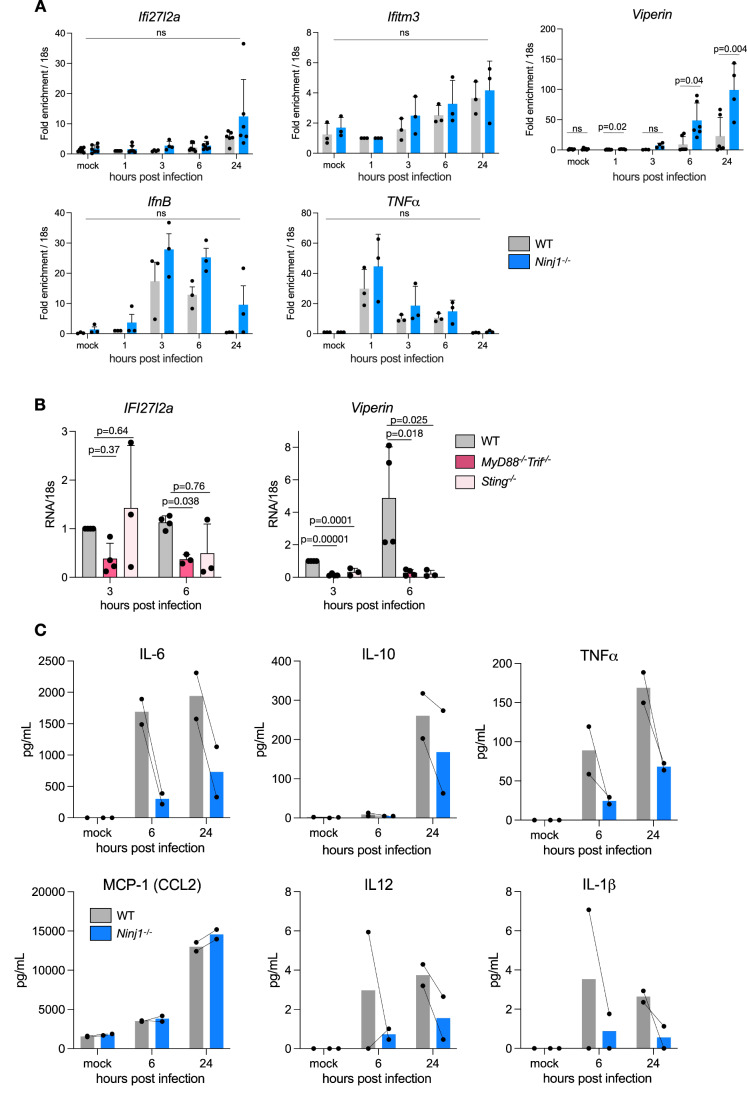
Loss of NINJ1 results in lower cytokine secretion. (**A**) WT and *Ninj1*^*–/–*^ iBMDMs were infected for the indicated times with HSV-1 GFP, and RNA levels of the indicated transcripts were measured by RT-qPCR. (**B**) WT, *MyD88*^*–/–*^*Trif*^*–/–*^, and *Sting*^*–/–*^ iBMDMs were infected with HSV-1 GFP for the indicated times, and RNA levels of the indicated transcripts were measured by RT-qPCR. (**C**) WT and *Ninj1*^*–/–*^ iBMDMs were infected for the indicated times with HSV-1 GFP, and supernatants were analyzed for the indicated cytokines. Data information: (**A**, **B**) mean ± SD is plotted with dots from biological replicates, (**A**) paired *t*-tests, ns not significant, *n* = 3–6. (**B**) one-way ANOVA with Dunnett’s multiple comparison test, *n* = 3–4, (**C**) bars show the mean and each dot represents a biological replicate (*n* = 2), lines show samples from experiments performed together. Source data are available online for this figure.

**Figure EV3:**
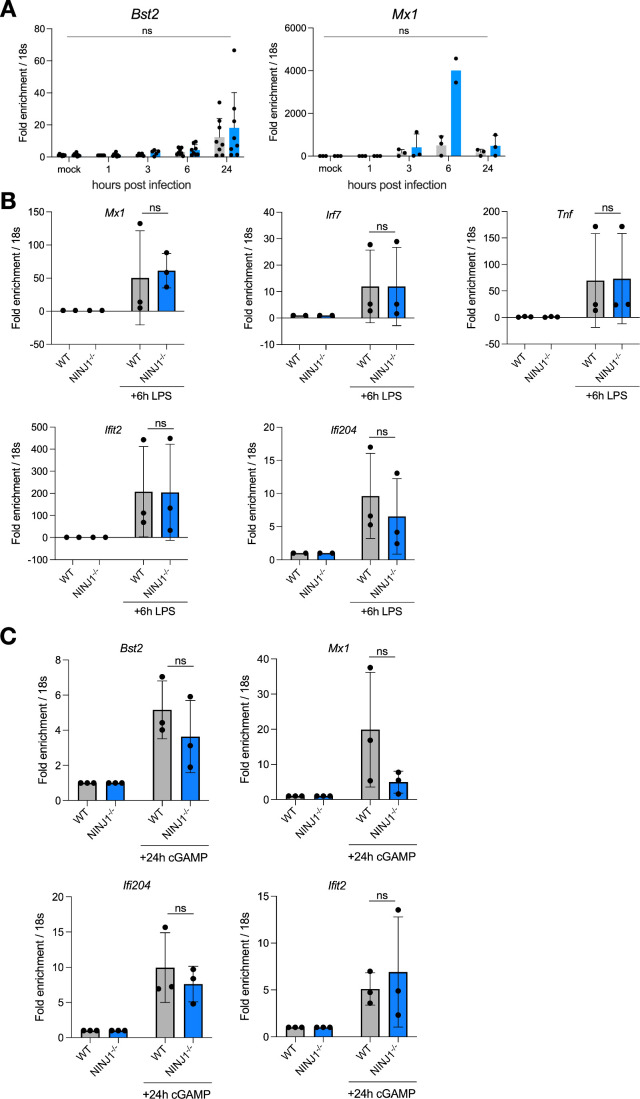
ISG transcript levels are higher in *Ninj1*^*−/−*^*cells* upon HSV-1 infection. (**A**) WT and *Ninj1*^*–/–*^ iBMDMs were infected for the indicated times with HSV-1 GFP, and RNA levels of the indicated transcripts were measured by RT-qPCR. (**B**) WT and *Ninj1*^*–/–*^ iBMDMs ± stimulation with 10 µg/mL LPS for 6 h. RNA was harvested and subjected to qPCR for the indicated transcripts. (**C**) WT and *Ninj1*^*–/–*^ iBMDMs ± stimulation with 5 µg/mL 2’3’cGAMP for 24 h. RNA was harvested and subjected to qPCR for the indicated transcripts. Data information: (**A**–**C**) mean ± SD is plotted with dots showing biological replicates, paired *t*-tests, ns not significant. (**A**) *n* = 3–8, (**B**, **C**) *n* = 3.

Given our observation that more viral particles enter *Ninj1*^*–/–*^ macrophages, we asked how this affects the innate immune response to HSV-1. We therefore measured the levels of several cytokines and ISGs in WT and *Ninj1*^*–/–*^ cells by RT-qPCR. We observed a trend of a stronger induction in *Ninj1*^*–/–*^ cells with the most pronounced difference in *Viperin* and *Ifnβ* expression and smaller differences in several other transcripts (Figs. 5A and EV3A). We thus asked what host pathway drives ISG induction in HSV-1-infected macrophages. Since TLRs and the cGAS/STING DNA sensing pathway have been implicated in the innate immune response to HSV-1 (Lima et al, 2010; Zhang et al, 2013; Reinert et al, 2016), we infected WT iBMDMs, *MyD88*^*–/–*^*Trif*^*–/–*^ cells, which abrogates signaling by TLRs, and *Sting*^*–/–*^ cells, which is needed for cGAS-mediated responses. Comparing the induction of ISGs at 3 hpi and 6 hpi, we observed the most marked decrease in *MyD88*^*–/–*^*Trif*^*–/–*^ cells (Fig. 5B). We also observed a decrease in *Sting*^*–/–*^ cells, but this was more consistent for the induction of *Viperin* as compared to the induction of *IFI2712a*. To check if TLR and cGAS/STING signaling was intact in WT and *Ninj1*^*–/–*^ cells, we stimulated WT and *Ninj1*^*–/–*^ cells with the TLR ligand lipopolysaccharide (LPS) and cGAMP (2’3’-cyclic GMP-AMP), the activator of the cGAS/STING pathway. We observed similar induction of ISGs after stimulation with both ligands, confirming that both pathways were functional and comparable in the two cell lines (Fig. EV3B,C). In summary, we implicate both TLR-mediated sensing and cytosolic DNA sensing in the macrophage response to HSV-1, and show that accelerated replication of HSV-1 in *Ninj1*-deficient cells results in a faster immune detection.

**Figure EV4:**
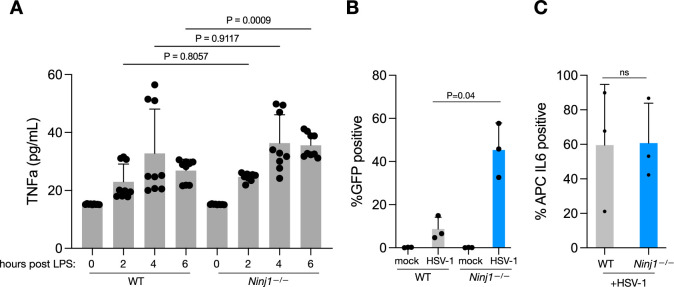
Loss of NINJ1 results in lower cytokine secretion. (**A**) WT and *Ninj1*^*–/–*^ iBMDMs were stimulated with 10 µg/mL LPS for the indicated periods of time. Supernatant were harvested and subjected to an ELISA for TNFα. Bars show the mean and SD. (**B**, **C**) WT and *Ninj1*^*–/–*^ iBMDMs were infected for 6 h and then stained for intracellular IL-6. Cells were analyzed on a flow cytometer for GFP levels (**B**) and APC IL-6 levels (**C**). Data information: (**A**–**C**) mean ± SD is plotted. A dots show three technical replicates from three biological replicates, Brown-Forsythe and Welch ANOVA. (**B**, **C**) dots show biological replicates, unpaired *t*-tests, ns not significant, *n* = 3.

Next, we asked if cytokine secretion followed the trend we observed in transcript levels of cytokines and ISGs. We thus performed a multiplex analysis of supernatants from HSV-1-infected WT and *Ninj1*^*–/–*^ macrophages. In striking contrast to the elevated transcript levels, we observed that *Ninj1*^*–/–*^ cells secreted three to six times less cytokines than WT cells (Fig. 5C). This difference was detectable for five out of six analyzed cytokines, namely for IL-6, IL-10, TNFα, IL-12 and IL-1β. To determine if this phenotype was specific to HSV-1 infection or if *Ninj1*-deficient cells had an intrinsic defect in cytokine secretion, we stimulated WT and *Ninj1*^*–/–*^ cells with LPS for 2, 4, and 6 h and measured the levels of TNFα in the supernatant by ELISA. We observed no defect in TNFα secretion at any timepoint in the *Ninj1*^*–/–*^ cells. Indeed, at 6 h post LPS stimulation, TNFα release was even slightly higher in *Ninj1*^*–/–*^ cells than in WT cells (Fig. EV4A). We thus reasoned that higher levels of HSV-1 gene expression could block secretion of cytokines in *Ninj1*^*–/–*^ cells, either by preventing their production or their release. To distinguish between these possibilities, we examined intracellular levels of IL-6 during HSV-1 infection, a cytokine that was detected to high levels in our multiplex analysis. We observed no defect in intracellular levels of IL-6 in the *Ninj1*^*–/–*^ cells despite detecting more infected cells (Fig. EV4B,C), suggesting that increased levels of viral replication prevent the secretion, rather than the production, of cytokines.

In summary, we report that NINJ1 acts as a gatekeeper for HSV-1 entry in mouse macrophages. We see that in the absence of NINJ1, a higher proportion of cells are infected with HSV-1, resulting in the production of more infectious virions. We specifically pinpoint the difference in infection rates to a difference in viral entry, with more viral particles entering *Ninj1*^*–/–*^ cells and a greater percentage of *Ninj1*^*–/–*^ cells getting infected. Thus, while NINJ1 has recently been identified as a key mediator of membrane rupture and cell lysis during cell death, we observe a novel, cell death-independent role for NINJ1 that renders macrophages refractory to HSV-1 infection at the site of first virus-cell contact. This work is in line with an intriguing novel hypothesis in the innate immune field, suggesting that the first line of defense against viral infection is PRR-independent and controls infection in the absence of generating an inflammatory response (Paludan et al, 2024).

Macrophages are recruited to the site of infection during both systemic (IV) and cutaneous mouse models of infection. During an encephalitis model, monocyte-like cells represented more than half of the total population harboring viral transcripts, indicating that HSV-1 enters these cells and that viral mRNA is produced (Ding et al, 2024). Mouse models of HSV-1 infection that lack macrophages succumb to disease much more quickly than controls, and microglia significantly attenuate the severity of CNS models of infection, highlighting the key role macrophages play in controlling lytic infection (Lang et al, 2020; Katzilieris-Petras et al, 2022). NINJ1 is highly expressed in macrophages and bone marrow-derived cells in comparison to most other cell types; however, the specific expression levels of NINJ1 in microglia of the brain has not been established. Furthermore, future work is required to understand the role of macrophages during human infection, the natural host of HSV-1. Here we observe that in the absence of NINJ1, ISG transcript expression increases, dependent on both TLR and cytosolic nucleic acid-dependent pathways. Surprisingly, despite transcript levels, infected *Ninj1*^*–/–*^ cells release less cytokines than WT cells. While this defect could be due to a low level of pore-forming activity of NINJ1 that we are unable to detect by crosslinking immunoblot, we disfavor this hypothesis as the examined cytokines are released by the canonical ER-Golgi secretion pathway (Stow et al, 2009). This distinguishes them from IL-1 family cytokines, including IL-1β, that are released through Gasdermin pores (Xia et al, 2021). In addition, stimulating cells with LPS showed comparable release of TNFα in the presence and absence of NINJ1, indicating no intrinsic defect in *Ninj1*^*–/–*^ cells for cytokine release. We thus favor a hypothesis where the loss of NINJ1 leads to more HSV-1 entry and increased viral gene expression that dampens cytokine secretion. Previous reports have found that HSV-1 suppresses pro-inflammatory cytokine expression, although these reports detected defects in cytokine RNA levels (Mogensen et al, 2004)

At the resting state, NINJ1 is known to exist as autoinhibited dimers (Pourmal et al, 2025), which we also observe. NINJ1 has further been described to maintain plasma membrane stability (Zhu et al, 2025). Thus, NINJ1 dimers at the resting state may change biophysical properties of the membrane, like fluidity, that have consequences for viral entry. In addition to being present on the plasma membrane, NINJ1 is also found on the nuclear membrane and in the secretory system (likely during trafficking to the plasma membrane)(Degen et al, 2023), and we cannot rule out secondary roles for NINJ1 during infection elsewhere in the cell. Furthermore, we do not observe oligomerization of NINJ1 during HSV-1 infection, even though we observe macrophages releasing LDH at late stages of infection, indicative of lytic cell death. While HSV-1 is known to be able to trigger apoptosis, necroptosis and pyroptosis (Aubert and Blaho, 2001; Guo et al, 2022; Parameswaran et al, 2024; Reinert et al, 2020), HSV-1 may directly block NINJ1 oligomerization. This could happen either by a direct viral protein-NINJ1 interaction or indirectly by viral-induced inhibition of cell death pathways that could then trigger NINJ1. Alternatively, LDH release could occur in a NINJ1-independent manner at late stages of infection.

In conclusion, we demonstrate a novel role for NINJ1 in preventing entry of HSV-1 in mouse macrophages, a cell type that expresses the greatest amount of NINJ1 in the body and is essential for HSV-1 infection.

## Methods

**Table:** Reagents and tools table

Reagent/resource	Reference or source	Identifier or catalog number
**Experimental models**		
HEK 293T cells (*H. sapiens*)	ATCC	CRL 3216
Vero cells (*C. aethiops*)	Florian Schmidt (Uni Bonn)	N/A
BSC-40 cells (*C. aethiops*)	Florian Schmidt (Uni Bonn)	N/A
Wildtype BMDM (*M. musculus*)	This study	N/A
*Ninj1−/−* BMDM (*M. musculus*)	This study	N/A
Wildtype iBMDM (*M. musculus*)	This study	N/A
*Ninj1−/−* iBMDM (*M. musculus*)	This study	N/A
MEF (*M. musculus*)	Dondelinger et al, (2023)	N/A
*Ninj1* ko MEF (*M. musculus*)	Dondelinger et al, (2023)	N/A
3T3 (*M. musculus*)	Ramos et al, (2024)	N/A
*Ninj1* ko 3T3 (*M. musculus*)	Ramos et al, (2024)	N/A
L929 (*M. musculus*)	ATCC	NCTN Clone 929
KOS HSV-1 ICP47 GFP	Florian Schmidt (Uni Bonn)	N/A
VACV Western Reserve EL EGFP	Florian Schmidt (Uni Bonn)	N/A
**Recombinant DNA**		
pLVX mNINJ1	Ramos et al, (2024)	Addgene 208775
pLJM1 Blasticidin	This paper	N/A
psPAX2	Addgene	12260
VSVG	Addgene	12259
**Antibodies**		
Mouse anti-ICP4	Santa Cruz	56986
Mouse anti-ICP5	Abcam	6508
Mouse anti-ICP8	Abcam	Ab20194
Rabbit anti-mNINJ1	Kayagaki et al, (2021)	N/A
Rabbit anti-hNINJ1	This study	N/A
Mouse anti-GAPDH	Santa Cruz	365062
HRP-anti-TUBULIN	Abcam	Ab40742
Rabbit anti-VINCULIN	Abcam	Ab91459
HRP-conjugated goat anti-mouse	Southern Biotech	1034-05
HRP-conjugated goat anti-rabbit	Southern Biotech	4030-05
Alexa Fluor-568 goat anti-mouse	Invitrogen	A11031
**Oligonucleotides and other sequence-based reagents**		
Ifi27I2a forward	GCAATTGCCAATGGAGGTGG	N/A
Ifi27I2a reverse	CCTGCGTAGTCTGTACAGGC	N/A
Bst2 forward	CGAGACACAGGCAAACTCCT	N/A
Bst2 reverse	CCAGAGTTCACCTGCACTGT	N/A
Viperin forward	TTGGGCAAGCTTGTGAGATTC	N/A
Viperin reverse	TGAACCATCTCTCCTGGATAAGG	N/A
Ifitm3 forward	GGTGGGTGATGTGACTGGAG	N/A
Ifitm3 reverse	GAAGCACTTCAGGACCGGAA	N/A
IfnB forward	CACAGCCCTCTCCATCAACT	N/A
IfnB reverse	GCATCTTCTCCGTCATCTCC	N/A
TNFa forward	TCTTCTCATTCCTGCTTGTGG	N/A
TNFa reverse	GGTCTGGGCCATAGAACTGA	N/A
Mx1 forward	ACCTGATGGCCTATCACCAG	N/A
Mx1 reverse	TTCAGGAGCCAGCTGTAGGT	N/A
Ifit2 forward	AGACAGTTACACAGCAGTCATGA	N/A
Ifit2 reverse	CACCCTGTCCTCAAACTCATC	N/A
Irf7 forward	CTGAAGTGAGGGGGGTCCAG	N/A
Irf7 reverse	CACAGCCCAGGCCTTGAAGA	N/A
18 s forward	GTAACCCGTTGAACCCCATT	N/A
18 s reverse	CCATCCAATCGGTAGTAGCG	N/A
**Chemicals, enzymes and other reagents**		
DMEM	Gibco	31966-021
Fetal calf serum (FCS)	Bioconcept	2-01F30-I
M-CSF	L929 cells	N/A
HEPES	Bioconcept	5-31F00-H
NEAA	Gibco	11140-035
L-glutamine	Gibco	25030-024
Sodium pyruvate	Gibco	11360070
Pen/Strep	BioConcept	4-01F00-H
DPBS	Gibco	14190-094
DPBS + CaCl_2_ + MgCl_2_	Gibco	14040-091
Trypsin	BioConcept	5-51F00-H
Opti-MEM	Gibco	51985-026
LT-1	Mirus	MIR2300
V-bottom 96-well analysis plate (TC treated)	Merck	P7116
Cell culture microplate, 96-well (TC treated)	TPP	92696
Cell culture microplate, 24-well (TC treated	TPP	92424
Cell culture microplate, 12-well (TC treated)	TPP	92412
Cell culture microplate, six-well (TC treated)	TPP	92406
Cell culture microplate, six-well (non TC treated)	Costar	2026-07-08
Petri dish, 10 cm	Falcon	351029
T75 flask	TPP	90076
T150 flask	TPP	90151
LDH	Roche	11644793001
BSA	Merck	A9647-
PAA	Merck	284270
Carboxymethylcellulose	Merck	C5678
Crystal Violet	Merck	V5265
4x Sample Buffer	Thermo Scientific	BN2003
DTT	Thermo Scientific	R0862
Trans-Blot Turbo 5x Transfer Buffer	Bio-Rad	10026938
Protease inhibitors	Merck	11836170001
Precast gels 4–20%	Millipore	MP41G10
Agarose	Merck	A9539
Nitrocellulose Membranes	Amersham	10600003
NaCl	Merck	1064045000
KCl	Merck	P4504
Citric acid	Merck	C0759
CellBrite 488	Biotium	BOT-3000-90-T
PFA	Electron Microscopy Science	15710
Saponin	Sigma	47036
Hoechst	Thermo Scientific	H3570
Dextran, 10000 MW	Thermo Scientific	P10361
Trizol	Thermo Scientific	15596026
Chloroform	Sigma Aldrich	C2432
Glycogen	Thermo Scientific	AM9510
Isopropanol	Merck	67-63-0
Ethanol	Merck	64-17-5
Methanol	Thermo Scientific	M/4000/17
DNase kit	Thermo Scientific	AM1907
MMLV	Life Technologies	28025013
RnaseOut RNase Inhibitor	Invitrogen	10777-019
SyberGreen Master Mix	Roche	04887352001
2’3’-cGAMP	Invivogen	Tlrl-nacga23-02
0111:B4 LPS	Invivogen	Tlrl-3pelps
Mouse TNF-alpha DuoSet ELISA	R&D systems	Dy410
APC mouse IL-6	BioLegend	504507
Annexin V APC	BioLegend	640920
Annexin V Binding Buffer	BioLegend	422201
Annexin V Staining Buffer	BioLegend	420201
Permeabilization Wash buffer	BioLegend	421002
Blasticidin	Invivogen	Ant-bl-1
Bs3	Thermo Scientific	21586
SYBR Safe DNA Gel Stain	Invivogen	S33102
0.45um filter	Millipore	SLHPR33RS
**Software**		
GraphPad Prism 10	www.graphpad.com	
ImageJ	Imagej.nih.gov/index/html	
SnapGene	Snapgene.com	
FlowJo	Flowjo.com	
**Other**		
LightCycler 480	Roche	
CytoFlex S	Beckman Coulter	
Zeiss LSM800 confocal	Zeiss	
TissueLyser	Qiagen	
Transblot Turbo	Bio-Rad	
iBright	Thermo Scientific	
Cytation5 Imaging Reader	BioTek	
Epoch Plate Reader	BioTek	

### Mammalian cell culture and generation

*WT* and *Ninj1*^*–/–*^ mouse bone marrow-derived macrophages (BMDMs) were harvested and differentiated in Dulbecco’s modified Eagle Medium (DMEM) (Gibco) containing 20% L929 supernatant as a source of macrophage colony-stimulating factor (M-CSF), 10% heat-inactivated fetal calf serum (FCS) (BioConcept), 10 mM Hepes (BioConcept), 1% penicillin/streptomycin, and 5 mL nonessential amino acids (NEAA, Gibco). Experiments were performed on days 9 to 10 of differentiation. Immortalization of macrophages was performed as previously described (Broz et al, 2010). Briefly, primary monocytes were extracted from bone marrow and then treated with filtered supernatant from existing iBMDMs for 24 h. Then these cells are grown for up to 2 months to wait for those that are immortalized to grow up. After several weeks in culture, M-CSF in the media is reduced from 20% to 10%. Immortalized macrophages (iBMDMs) were cultured in DMEM containing 10% FCS, 10% M-CSF, 10 mM Hepes, and 5 mL nonessential amino acids. HEK 293T and 3T3 cells were cultured in DMEM supplemented with 10% FCS. 3T3 KO and controls have been previously described (Ramos et al, 2024). MEFs were grown in DMEM with 10% FCS, 2 mM L-glutamine, and 400 µM sodium pyruvate and were previously published (Dondelinger et al, 2023). All cells were grown at 37 °C, 5% CO_2_. Cells are regularly tested for mycoplasma.

### Viral propagation, infection, and plaque assays

KOS HSV-1 GFP (ICP47 promoter drives GFP, a kind gift of Florian Schmidt) was propagated on Vero cells. Briefly, 90% confluent cells were infected at an MOI of 0.01 and left to grow for 3–4 days. Cell free viral particles were combined with cells, subjected to douncing, and spun at 500×*g* for 5 m. The supernatant was then ultracentrifuged at 15,000×*g* for 90 min, and the viral pellet was resuspended in DMEM with 10% FCS and 1 g/100 mL of BSA. Viral preps were titered on Vero cells, and MOIs reflect Vero cell numbers. Plaque assays were performed by seeding Vero cells at 1 × 10^4^ cells per well in 24-well plates. The next day, cells were infected in 500 uL for 2 h, then aspirated and replaced with full media supplemented with 0.5% carboxymethylcellulose, then left to grow for 3 days. Cells were fixed with 4% paraformaldehyde for 10 min, then aspirated and stained with 0.5% crystal violet for 10 m before counting.

Infections were performed at an MOI of 10 when not indicated otherwise. For macrophage infections, viral supernatants were left on cells for 3 h in opti-MEM before changing media, except for experiments analyzed by BioTek Cytation5 plate reader, where the virus was added to cells and not removed.

VACV (Western Reserve EL EGFP, a kind gift of Florian Schmidt) was propagated on BSC-40 cells. Briefly, two 90% confluent T150 flasks were infected at MOI 0.01 and 2 days later, scraped and subjected to two freeze-thaw cycles. Cellular debris was centrifuged out at 300×*g* for 10 min, and the supernatant was centrifuged at 15,000×*g* for 90 min. The viral pellet was resuspended in DMEM and titered on BSC-40 cells.

### Immunoblotting

For western blotting analysis, cells were lysed in 66 mM Tris-HCl, pH 7.4, 2% SDS, 10 mM DTT, and NuPage LDS sample buffer (Thermo Fisher). For organ samples, 30 mg of each organ was homogenized in 300 uL of RIPA buffer with protease inhibitors (Roche) in a TissueLyser (Qiagen) with a metal bead for 2 × 2 m at 30 Hz. Lysates were then spun at top speed for 3 min, and the supernatant was used for blotting. Cell lysates were separated on gradient precast gels 4–20% (Milipore) and transferred to a nitrocellulose membrane using Transblot Turbo (Bio-Rad). The antibodies used were: anti-ICP4 (Santa Cruz 56986), anti-ICP8 (Abcam 20194), anti-gD (Santa Cruz 69802) anti-mouse NINJ1 (rabbit IgG2b clone 25; a kind gift from Genentech; 1:8000), anti-human NINJ1 (commercially generated for this publication by Yenzym, 1:500), anti-tubulin (ab40742; Abcam; 1:2000), anti-GAPDH (365062; Santa cruz, 1:3000), anti-VINCULIN (Ab91459; Abcam; 1:1000). Primary antibodies were detected with horseradish peroxidase (HRP)-conjugated goat anti-rabbit (4030-05; Southern Biotech; 1:5000), HRP-conjugated goat anti-mouse (1034-05; Southern Biotech; 1:5000 or 12-349; MilliporeSigma; 1:2000) secondary antibodies.

### Complementation of NINJ1 in *Ninj1*^*–/–*^ iBMDMs

pLVX mNINJ1 (addgene 208775) was made into a lentivirus by transfecting 3 million 293T cells in a six-well dish with 1.25 µg of packaging plasmid (pLVX mNINJ1), 1.25 µg of psPAX2 and 250 ng of VSVG and 8 uL LT-1 transfection reagent (Mirus). Media was changed 24 h post transfection to DMEM + 10% FCS + 1 g/100 mL bovine serum albumin (BSA), lentivirus was harvested 48 h post transfection and filtered through a 0.45 µm filter. 1.5 million iBMDMs were transduced with 1 mL of virus by spinning at 500×*g* for 1.5 h in one well of a non-tissue culture-treated six-well dish. Next, cells were split out into a 10 cm petri dish and selected with 1 µg/mL puromycin 24 h post transduction for at least 5 days. For transgene-expressing experiments, a pLJM1 vector carrying blasticidin resistance was packaged into lentivirus as described above for complementation and spinfected into *Ninj1*^*–/–*^ cells as described above. Cells were then selected with 5 µg/mL Blasticidin for 5 days before experiments were performed, infecting cells ± the Blasticidin resistance cassette with the same volume of HSV-1.

### Entry experiments

For infections where entry by endocytosis was quantified, after addition of viral supernatants onto cells, cells were washed with a low pH citric acid buffer (135 mM NaCl, 10 mM KCl, 40 mM citric acid, pH 3) for 5 m to inactivate any extracellular viral particles.

### Annexin V flow cytometry

Macrophages were seeded in tissue culture-treated 12-well plates at 1 × 10^5^ cells/well. Supernatant and adherent cells were combined by trypsinizing adherent cells and then staining with Annexin V APC (BioLegend) following the manufacturer’s protocol, using BioLegend Cell Staining Buffer and Annexin V Binding Buffer (BioLegend). Cells were analyzed on a Cytoflex S. All flow cytometry analysis was performed in FloJo.

### Cell lysis assays

A day before stimulation, cells were seeded in 96-well plates at a density of 2 × 10^4^ iBMDMs per well. Cells were infected at an MOI of 10. Cell lysis was quantified by measuring the lactate dehydrogenase (LDH) amount in the cell supernatant using the LDH cytotoxicity kit (Takara, Clontech) according to the manufacturer’s instructions and expressed as a percentage of total LDH release. LDH release was normalized to untreated control and 100% lysis control, by adding Triton X-100 to a final concentration of 0.01% as follows: (LDH_sample_ – LDH_negative control_)/(LDH_100% lysis_ – LDH_negative control_) × 100.

### Crosslinking assay

WT BMDMs were seeded in 24-well plates at a density of 2 × 10^5^ cells per well a day before stimulation. The next day, cells were infected with HSV-1 at an MOI of 50 for 3 h, or the relevant time post infection. Cells were washed once in Ca^2+^Mg^2+^ containing PBS, and then 200 uL of Ca^2+^Mg^2+^ containing PBS was left on the cells. Next, the crosslinker BS^3^ (bis(sulfosuccinimidyl)suberate) was added according to the manufacturer’s instructions. In brief, BS^3^ was added to the media (3 mM; Thermo Fisher) and incubated for 5 m at room temperature. Next, a solution of 20 mM Tris pH 7.5 was added to stop the crosslinking reaction and incubated for 10 m at room temperature. Cell supernatants were collected, and proteins were precipitated with methanol and chloroform and combined with cell lysates for western blotting analysis.

### Confocal microscopy and image analysis

iBMDMs seeded onto glass coverslips were infected for 45 m at an MOI of 10. For the last 5 m of infection, they were treated with CellBrite (Biotium) to label the plasma membrane. Then cells were fixed in 4% PFA for 10 min, washed with PBS, permeabilized with 0.05% saponin and blocked with 1% BSA in PBS. Then, samples were incubated with an anti-ICP5 antibody (Abcam 6508), then an Alexa Fluor-568 conjugated antibody and Hoechst (1:1000). Samples were then imaged with a Zeiss LSM800 confocal laser scanning microscope using a 63x/1.4 NA oil objective. Quantification of capsids was performed using a maximum projection of the z-stack, by manually segmenting cells and counting the number of infected cells per field of view and the number of capsids per cell. All microscopy datasets were analyzed and processed using Fiji software. Raw microscopy images are available on BioImage Archive: 10.6019/S-BIAD2327.

### Dextran uptake assays

xiBMDMs were seeded at 5 × 10^5^ in 100 uL of FACS buffer (PBS with 2% FCS and 3 mM EDTA) in a V-bottom 96-well plate. Fluorescent dextran was added to a final concentration of either 0.05 uM or 0.1 uM and incubated at 37 °C for 30 m with gentle agitation every 10 min. Each well was then supplemented with 100 uL FACS buffer, spun at 300×*g* for 5 m at 4 °C, washed once with 200 uL FACS buffer, spun again as before and resuspended in 250 uL FACS buffer for analysis. After the initial 30 min incubation, samples were kept on ice to prevent additional dextran entry until being analyzed by flow cytometry.

### Antibody generation

Rabbit anti-human NINJ1 was made by YenZym antibodies, LLC to a synthetic NINJ1 peptide comprising the first 76 aa of human NINJ1 (GenScript).

### RT-qPCR

RNA was isolated with Trizol. Briefly, 1 × 10^5^ cells seeded the day before and then infected were washed and resuspended in 50 uL of PBS, then lysed in 500 uL of Trizol. About 100 uL of chloroform was added and spun for 15 m at 21,000×*g* at 4 °C. The top phase was moved into a new tube with 5 uL of glycogen (Invitrogen) and 500 uL of isopropanol. After 10 min at room temperature, samples were spun again for 10 m at 21,000×*g* at 4 °C, and then washed twice with 70% ethanol, spinning for 5 min after each spin. Then ethanol was removed, samples were resuspended in Rnase/Dnase-free water. 1.5 ug of RNA was DNase-treated (Thermo DNA-free Kit) and then subjected to RT with MMLV following the manufacturer’s guidelines. RT-qPCR was performed with SybrGreen Master Mix on a LightCycler 480 (Roche). Primers used for RT-qPCR are listed in the Reagents and Tools Table.

### LPS and cGAMP stimulation

Cells were stimulated with 5 µg/mL 2’3’ cGAMP (Invivogen) or 10 µg/mL 0111:B4 LPS (Invivogen) by adding the appropriate concentration to total media for the indicated amounts of time.

### Animals and primary cells

All experiments involving animals were performed under the guidelines and approval from the cantonal veterinary office of the canton of Vaud (Switzerland), license number VD3895. All mice were bred and housed at a specific-pathogen-free facility at 22 C° room temperature, 10% humidity and a day/night cycle of 12 h/12 h at the University of Lausanne. *Ninj1*-deficient mice have been described before (Degen et al). *Myd88*^*–/–*^*Sting*^*–/–*^, *Sting*^*–/–*^, *Ripk3*^–/–^, *Pycard*^–/–^ (ASC), and caspase-3 KO cells have been described before (Heilig et al, 2020; Mariathasan et al, 2004). Animals used in the study were between 8 and 10 weeks old.

### Cytokine analysis

WT and *Ninj1*^–/–^ immortalized BMDMs were infected for 3 h in opti-MEM, and then the media was changed to complete media. Infections were left for a total of 6 or 24 h, and then the supernatant was harvested and treated with 0.1% Triton X-100 to inactivate any viral particles. Eve Technologies (Calgary, Canada) performed the cytokine analysis shown in Fig. 5C.

### ELISA

Mouse TNF-alpha DuoSet ELISA (R&D systems DY410) was performed on 75 µL of supernatant (from a total of 200 µL) from 50,000 cells seeded at the time of stimulation in a 96-well plate. Each condition was performed in technical triplicate. ELISA was performed following the manufacturer’s protocol.

### Intracellular IL-6 staining

Cells were seeded at 400,000 per well of a six-well dish and infected in Opti-MEM for 6 h. Cells were then scraped, fixed in 4% PFA for 10 m and permeabilized with BioLegend Intracellular Staining Permeabilization Wash Buffer, following the manufacturer’s recommendations. APC Mouse IL-6 was then incubated for 1 h at RT, and samples were analyzed on a flow cytometer.

## Supplementary information

Peer Review File

Source data Fig. 1

Source data Fig. 2

Source data Fig. 3

Source data Fig. 4

Source data Fig. 5

Expanded View Figures

## Data Availability

Microscopy data related to Fig. 3H from this study is available on BioImage Archive: https://www.ebi.ac.uk/biostudies/bioimages/studies/S-BIAD2327 The source data of this paper are collected in the following database record: biostudies:S-SCDT-10_1038-S44319-025-00638-8.

## References

[CR1] Aubert M, Blaho JA (2001) Modulation of apoptosis during herpes simplex virus infection in human cells. Microbes Infect 3:859–866 11580981 10.1016/s1286-4579(01)01444-7

[CR2] Borges JP, Sætra RS, Volchuk A, Bugge M, Devant P, Sporsheim B, Kilburn BR, Evavold CL, Kagan JC, Goldenberg NM et al (2022) Glycine inhibits NINJ1 membrane clustering to suppress plasma membrane rupture in cell death. Elife 11:e78609 36468682 10.7554/eLife.78609 PMC9754625

[CR3] Broz P, von Moltke J, Jones JW, Vance RE, Monack DM (2010) Differential requirement for caspase-1 autoproteolysis in pathogen-induced cell death and cytokine processing. Cell Host Microbe 8:471–483 21147462 10.1016/j.chom.2010.11.007 PMC3016200

[CR4] Degen M, Santos JC, Pluhackova K, Cebrero G, Ramos S, Jankevicius G, Hartenian E, Guillerm U, Mari SA, Kohl B et al (2023) Structural basis of NINJ1-mediated plasma membrane rupture in cell death. Nature 618:1065–1071 37198476 10.1038/s41586-023-05991-z PMC10307626

[CR5] Ding X, Lai X, Klaestrup IH, Jensen SRN, Nielsen MM, Thorsen K, Romero-Ramos M, Luo Y, Lin L, Reinert LS et al (2025) Temporally resolved single-cell RNA sequencing reveals protective and pathological responses during herpes simplex virus CNS infection. J Neuroinflammation 22:146 40450318 10.1186/s12974-025-03471-x PMC12125739

[CR6] Ding X, Lai X, Klæstrup IH, Jensen SRN, Nielsen MM, Thorsen K, Romero-Ramos M, Luo Y, Lin L, Reinert LS et al (2024) Temporally resolved single-cell RNA sequencing reveals protective and pathological responses during herpes simplex virus CNS infection. J Neuroinflammation 22:146 10.1186/s12974-025-03471-x PMC12125739 40450318

[CR7] Dondelinger Y, Priem D, Huyghe J, Delanghe T, Vandenabeele P, Bertrand MJM (2023) NINJ1 is activated by cell swelling to regulate plasma membrane permeabilization during regulated necrosis. Cell Death Dis 14:755 37980412 10.1038/s41419-023-06284-z PMC10657445

[CR8] Guo H, Gilley RP, Fisher A, Lane R, Landsteiner VJ, Ragan KB, Dovey CM, Carette JE, Upton JW, Mocarski ES et al (2018) Species-independent contribution of ZBP1/DAI/DLM-1-triggered necroptosis in host defense against HSV1. Cell Death Dis 9:816 30050136 10.1038/s41419-018-0868-3 PMC6062522

[CR9] Guo H, Koehler HS, Mocarski ES, Dix RD (2022) RIPK3 and caspase 8 collaborate to limit herpes simplex encephalitis. PLoS Pathog 18:e1010857 36121858 10.1371/journal.ppat.1010857 PMC9521923

[CR10] Hartenian E, Bernard EM, Ammirati G, Monnier S, Leloup HB, Agustoni M, Bissegger L, Santos JC, Mari S, Sieben C et al (2024) Ninjurin-1 mediated plasma membrane r upture is a two-step process requiring cell swelling. Preprint at bioRxiv 10.1101/2024.10.29.620849

[CR11] Heilig R, Dilucca M, Boucher D, Chen KW, Hancz D, Demarco B, Shkarina K, Broz P (2020) Caspase-1 cleaves Bid to release mitochondrial SMAC and drive secondary necrosis in the absence of GSDMD. Life Sci Alliance 3: e202000735 32345661 10.26508/lsa.202000735 PMC7190276

[CR12] Kaiser WJ, Upton JW, Mocarski ES (2013) Viral modulation of programmed necrosis. Curr Opin Virol 3:296–306 23773332 10.1016/j.coviro.2013.05.019 PMC3821070

[CR13] Katzilieris-Petras G, Lai X, Rashidi AS, Verjans GMGM, Reinert LS, Paludan SR (2022) Microglia activate early antiviral responses upon herpes simplex virus 1 entry into the brain to counteract development of encephalitis-like disease in mice. J Virol 96:e01311–e01321 35045263 10.1128/jvi.01311-21 PMC8941881

[CR14] Kayagaki N, Kornfeld OS, Lee BL, Stowe IB, O’Rourke K, Li Q, Sandoval W, Yan D, Kang J, Xu M et al (2021) NINJ1 mediates plasma membrane rupture during lytic cell death. Nature 591:131–136 33472215 10.1038/s41586-021-03218-7

[CR15] Lang J, Bohn P, Bhat H, Jastrow H, Walkenfort B, Cansiz F, Fink J, Bauer M, Olszewski D, Ramos-Nascimento A et al (2020) Acid ceramidase of macrophages traps herpes simplex virus in multivesicular bodies and protects from severe disease. Nat Commun 11:1338 32165633 10.1038/s41467-020-15072-8 PMC7067866

[CR16] Lee H-J, Ahn BJ, Shin MW, Choi J-H, Kim K-W (2010) Ninjurin1: a potential adhesion molecule and its role in inflammation and tissue remodeling. Mol Cells 29:223–227 20119872 10.1007/s10059-010-0043-x

[CR17] Lima GK, Zolini GP, Mansur DS, Lima BHF, Wischhoff U, Astigarraga RG, Dias MF, Silva MdasGA, Béla SR, Antonelli LR, do V et al (2010) Toll-like receptor (TLR) 2 and TLR9 expressed in trigeminal ganglia are critical to viral control during herpes simplex virus 1 infection. Am J Pathol 177:2433–2445 20864677 10.2353/ajpath.2010.100121 PMC2966801

[CR18] Mariathasan S, Newton K, Monack DM, Vucic D, French DM, Lee WP, Roose-Girma M, Erickson S, Dixit VM (2004) Differential activation of the inflammasome by caspase-1 adaptors ASC and Ipaf. Nature 430:213–218 15190255 10.1038/nature02664

[CR19] Maruzuru Y, Ichinohe T, Sato R, Miyake K, Okano T, Suzuki T, Koshiba T, Koyanagi N, Tsuda S, Watanabe M et al (2018) Herpes simplex virus 1 VP22 inhibits AIM2-dependent inflammasome activation to enable efficient viral replication. Cell Host Microbe 23:254–265.e7 29447697 10.1016/j.chom.2017.12.014

[CR20] Melchjorsen J, Sirén J, Julkunen I, Paludan SR, Matikainen S (2006) Induction of cytokine expression by herpes simplex virus in human monocyte-derived macrophages and dendritic cells is dependent on virus replication and is counteracted by ICP27 targeting NF-κB and IRF-3. J Gen Virol 87:1099–1108 16603509 10.1099/vir.0.81541-0

[CR21] Mogensen TH, Melchjorsen J, Malmgaard L, Casola A, Paludan SR (2004) Suppression of proinflammatory cytokine expression by herpes simplex virus type 1. J Virol 78:5883–5890 15140986 10.1128/JVI.78.11.5883-5890.2004 PMC415838

[CR22] Paludan SR, Pradeu T, Pichlmair A, Wray KB, Mikkelsen JG, Olagnier D, Mogensen TH (2024) Early host defense against virus infections. Cell Rep 43:115070 39675007 10.1016/j.celrep.2024.115070

[CR23] Parameswaran P, Payne L, Powers J, Rashighi M, Orzalli MH (2024) A viral E3 ubiquitin ligase produced by herpes simplex virus 1 inhibits the NLRP1 inflammasome. J Exp Med 221:e20231518 38861480 10.1084/jem.20231518 PMC11167375

[CR32] Pourmal S, Truong ME, Johnson MC, Yang Y, Zhou L, Alegre K, Stowe IB, Gupta S, Chen PA, Zhang Y, Rohou A, Newton K, Kayagaki N, Dixit VM, Deshpande I (2025) Autoinhibition of dimeric NINJ1 prevents plasma membrane rupture. Nature 637:446–452 10.1038/s41586-024-08273-4 PMC11711097 39476863

[CR24] Ramos S, Hartenian E, Santos JC, Walch P, Broz P (2024) NINJ1 induces plasma membrane rupture and release of damage-associated molecular pattern molecules during ferroptosis. EMBO J 43:1164–1186 38396301 10.1038/s44318-024-00055-y PMC10987646

[CR25] Reinert LS, Lopušná K, Winther H, Sun C, Thomsen MK, Nandakumar R, Mogensen TH, Meyer M, Vægter C, Nyengaard JR et al (2016) Sensing of HSV-1 by the cGAS–STING pathway in microglia orchestrates antiviral defence in the CNS. Nat Commun 7:13348 27830700 10.1038/ncomms13348 PMC5109551

[CR26] Reinert LS, Rashidi AS, Tran DN, Katzilieris-Petras G, Hvidt AK, Gohr M, Fruhwürth S, Bodda C, Thomsen MK, Vendelbo MH et al (2020) Brain immune cells undergo cGAS-STING-dependent apoptosis during herpes simplex virus type 1 infection. J Clin Investig 131:e136824 10.1172/JCI136824 PMC7773356 32990676

[CR27] Stow JL, Low PC, Offenhäuser C, Sangermani D (2009) Cytokine secretion in macrophages and other cells: Pathways and mediators. Immunobiology 214:601–612 19268389 10.1016/j.imbio.2008.11.005

[CR28] Wang G, Zhang D, Orchard RC, Hancks DC, Reese TA (2023) Norovirus MLKL-like protein initiates cell death to induce viral egress. Nature 616:152–158 10.1038/s41586-023-05851-w PMC10348409 36991121

[CR29] Xia S, Zhang Z, Magupalli VG, Pablo JL, Dong Y, Vora SM, Wang L, Fu T-M, Jacobson MP, Greka A et al (2021) Gasdermin D pore structure reveals preferential release of mature interleukin-1. Nature 593:607–611 33883744 10.1038/s41586-021-03478-3 PMC8588876

[CR30] Zhang S-Y, Herman M, Ciancanelli MJ, de Diego RP, Sancho-Shimizu V, Abel L, Casanova J-L (2013) TLR3 immunity to infection in mice and humans. Curr Opin Immunol 25:19–33 23290562 10.1016/j.coi.2012.11.001 PMC3594520

[CR31] Zhu Y, Xiao F, Wang Y, Wang Y, Li J, Zhong D, Huang Z, Yu M, Wang Z, Barbara J et al (2025) NINJ1 regulates plasma membrane fragility under mechanical strain. Nature 644:1088–1096 40490006 10.1038/s41586-025-09222-5 PMC12210241

