# Supplementary figures and images for: NINJ1 blocks HSV-1 entry into macrophages to impact viral replication and immunity
Source: EMBO Rep. 2025 Nov 19;27(1):69–88. doi: 10.1038/s44319-025-00638-8 (PMC12796307; doi:10.1038/s44319-025-00638-8)

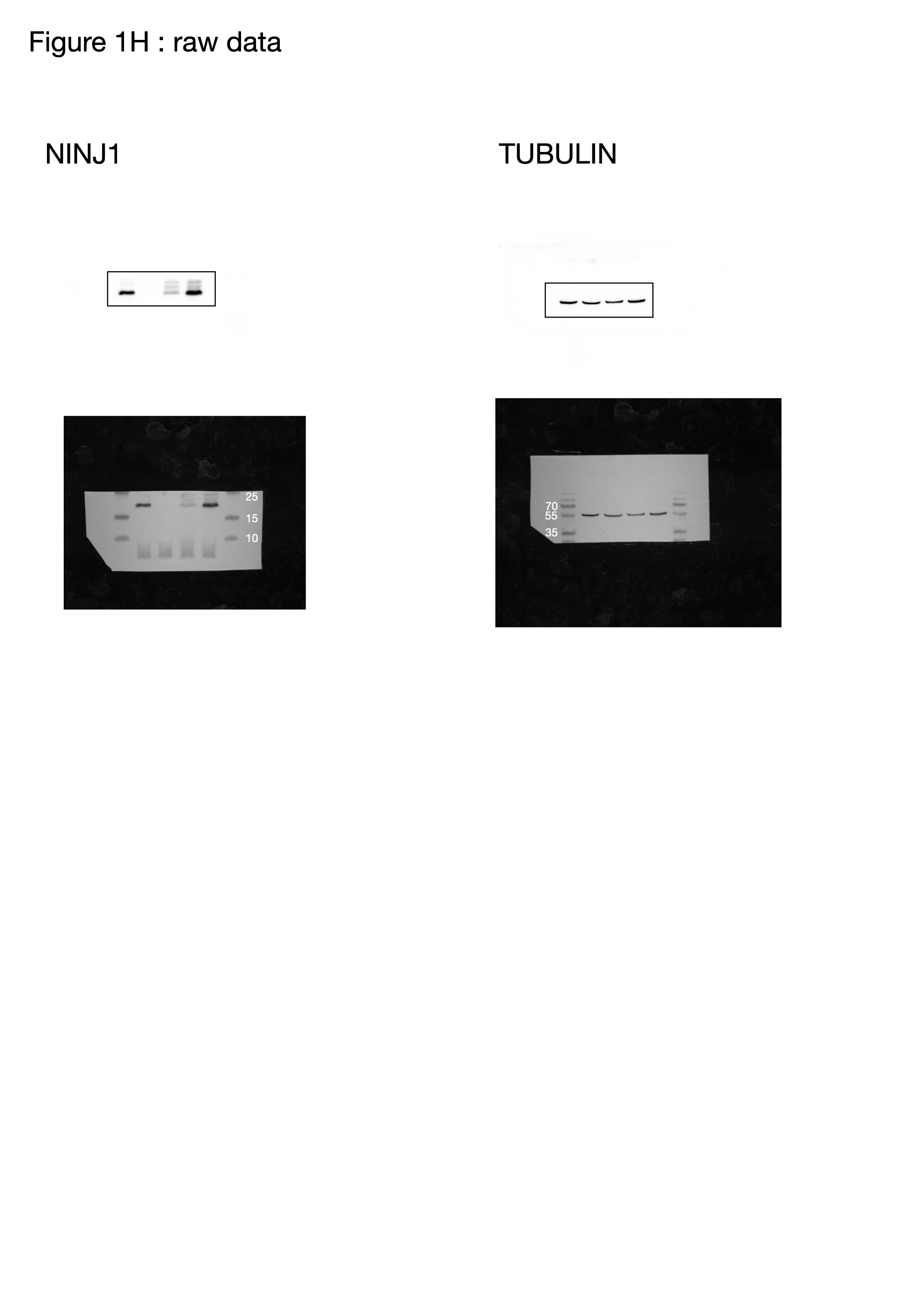

Supplement: Supplementary file 2 — Source data Fig. 1 [file 44319_2025_638_MOESM2_ESM.zip › Figure 1/Fig1H.tiff]

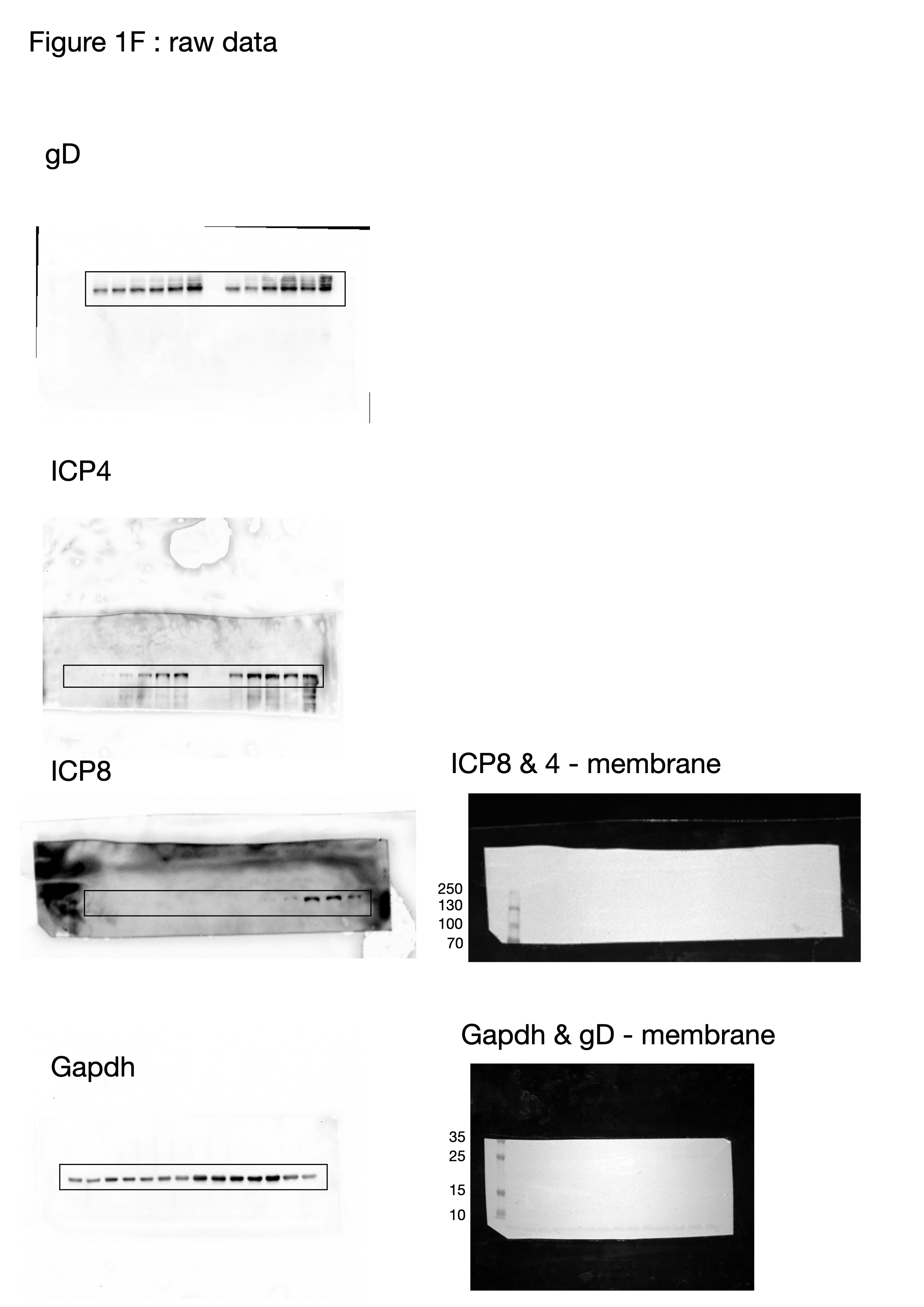

Supplement: Supplementary file 2 — Source data Fig. 1 [file 44319_2025_638_MOESM2_ESM.zip › Figure 1/Fig1F.tiff]

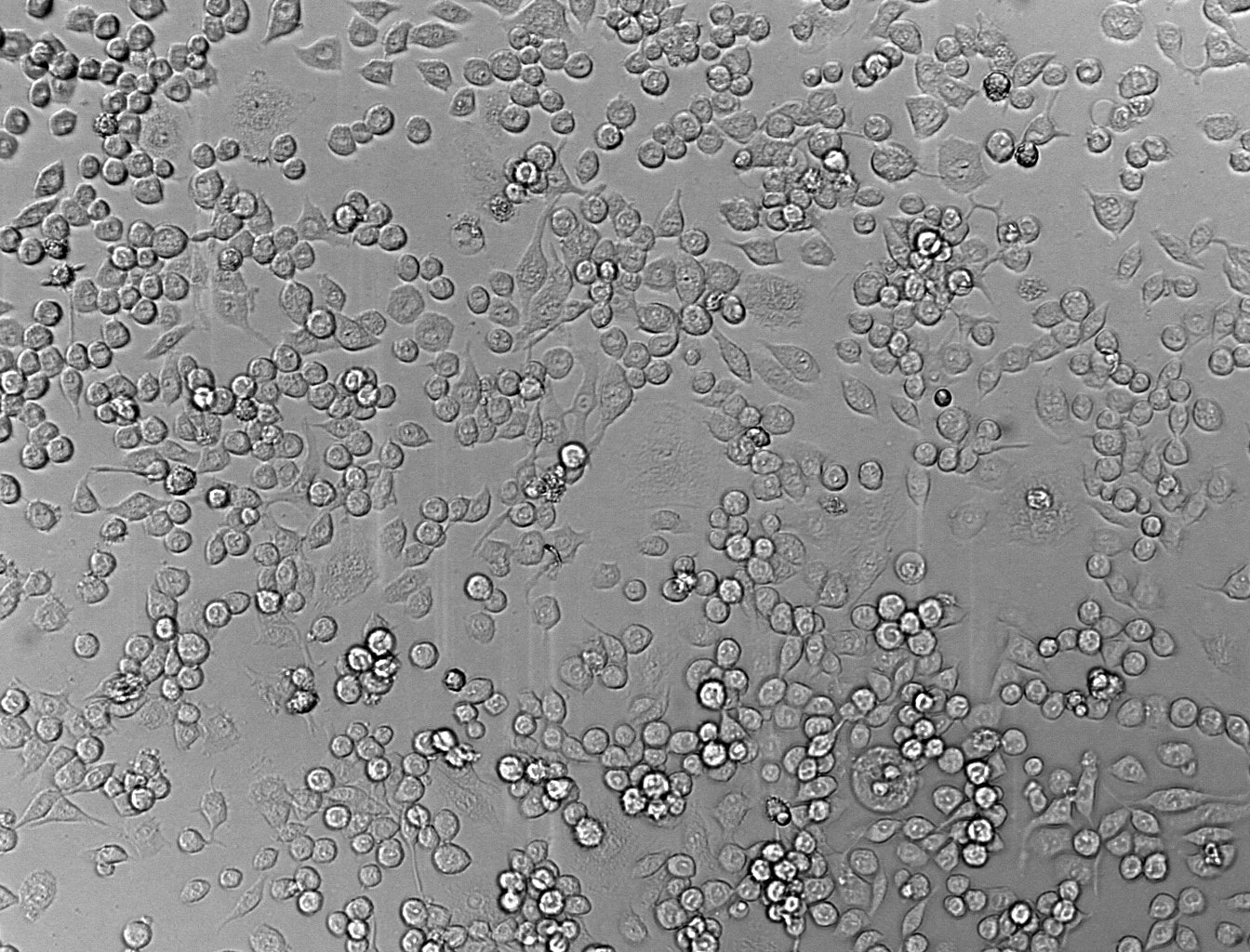

Supplement: Supplementary file 2 — Source data Fig. 1 [file 44319_2025_638_MOESM2_ESM.zip › Figure 1/Fig1C/NINJ1 ko Bright field.jpg]

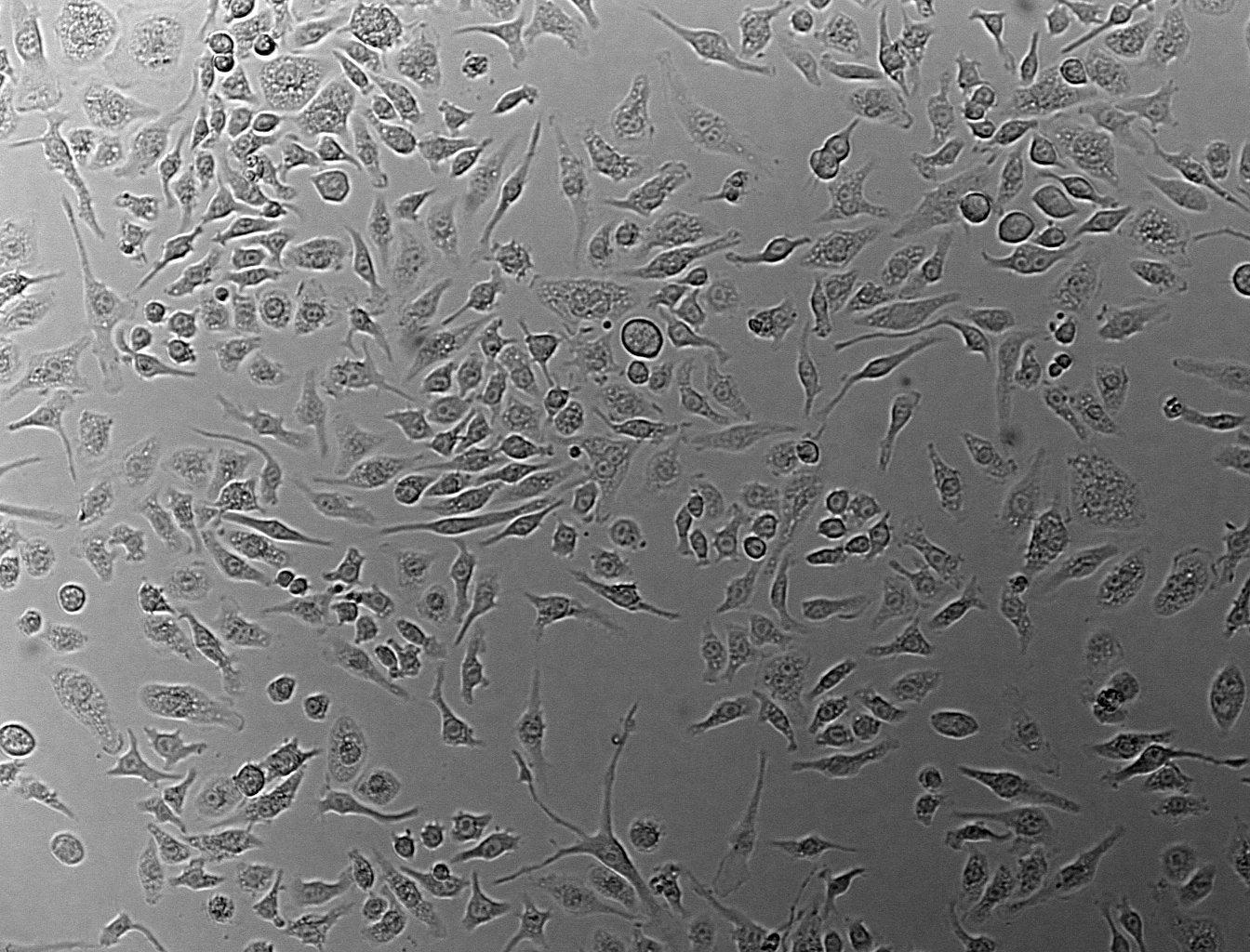

Supplement: Supplementary file 2 — Source data Fig. 1 [file 44319_2025_638_MOESM2_ESM.zip › Figure 1/Fig1C/WT bright field.jpg]

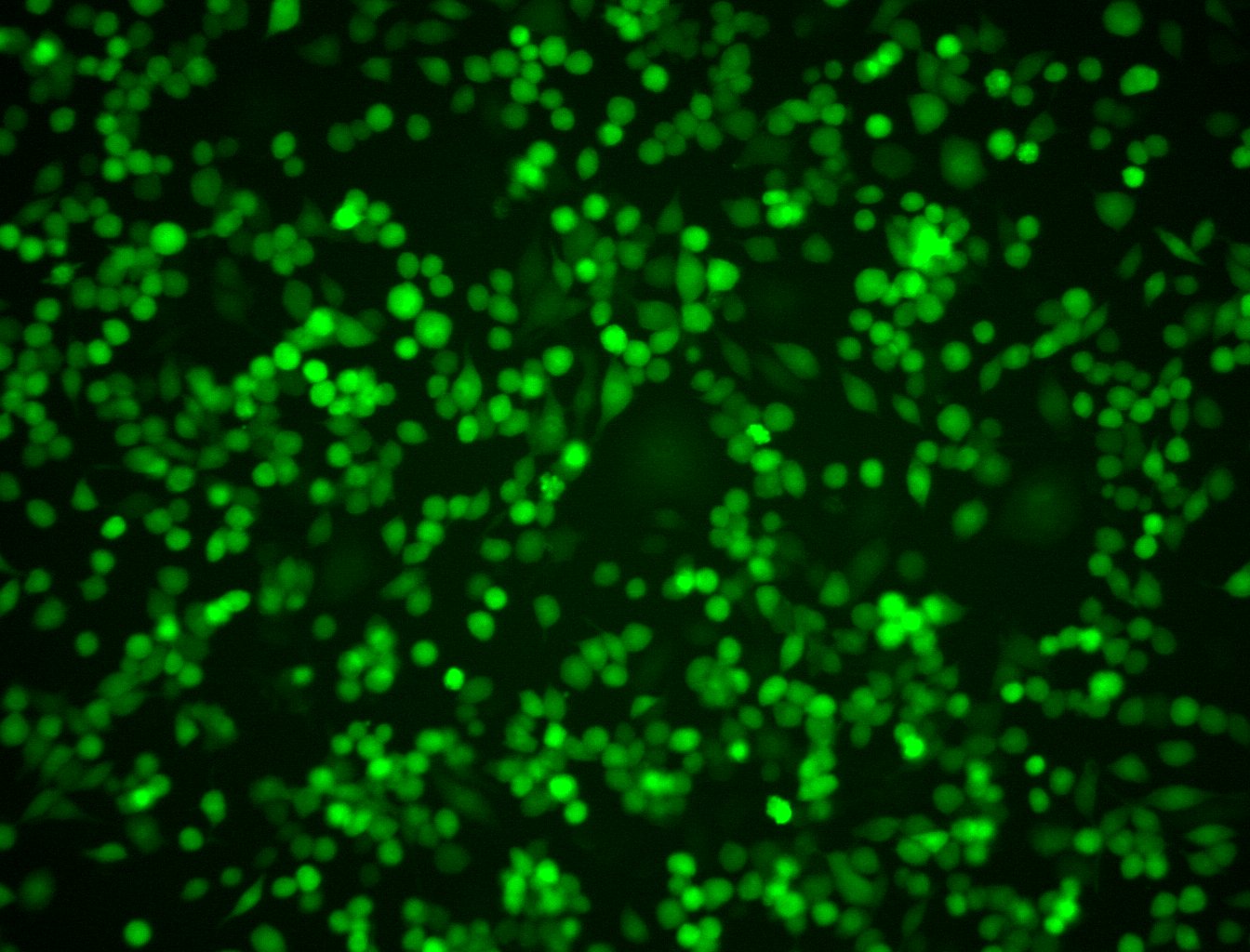

Supplement: Supplementary file 2 — Source data Fig. 1 [file 44319_2025_638_MOESM2_ESM.zip › Figure 1/Fig1C/NINJ1 ko GFP.jpg]

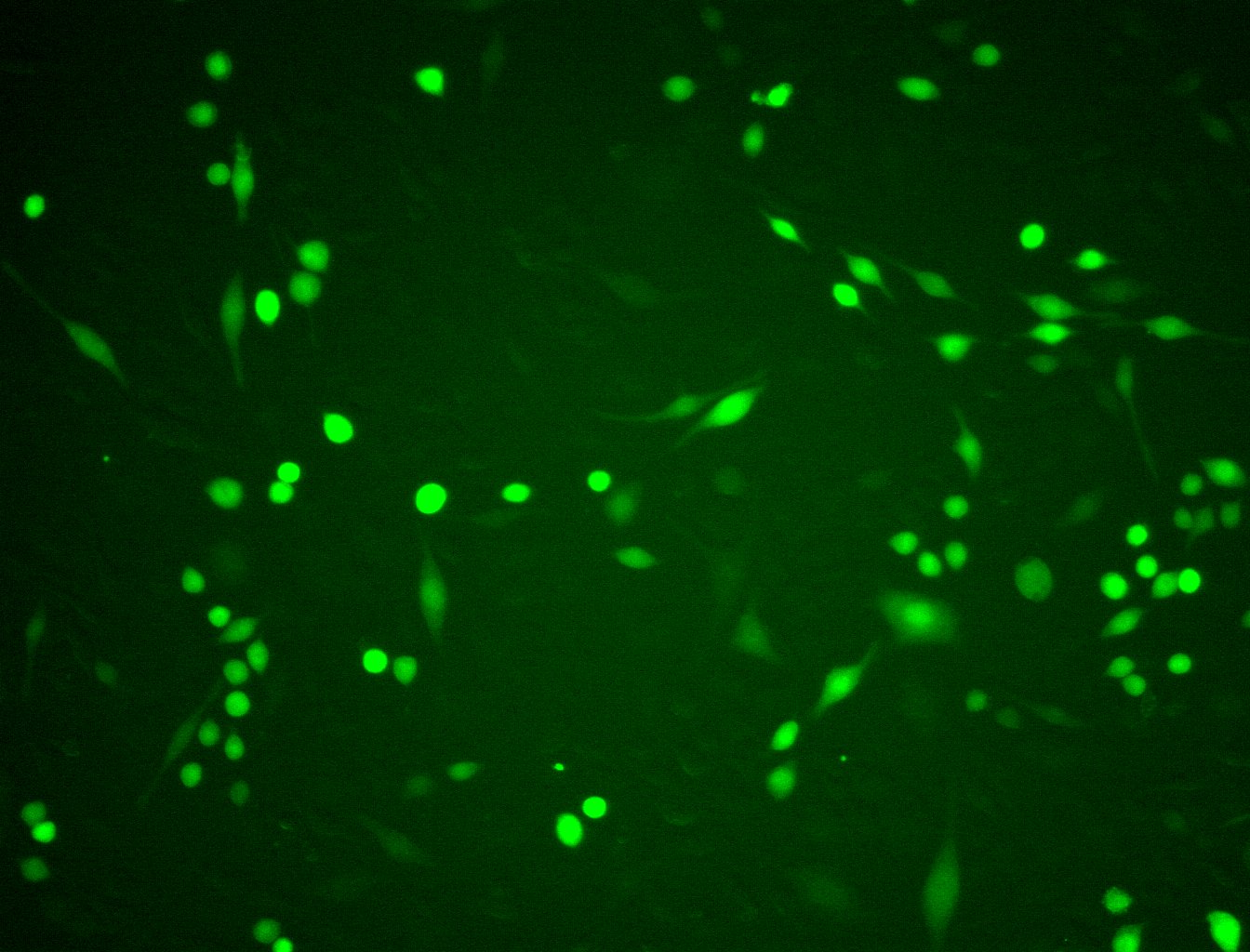

Supplement: Supplementary file 2 — Source data Fig. 1 [file 44319_2025_638_MOESM2_ESM.zip › Figure 1/Fig1C/WT GFP.jpg]

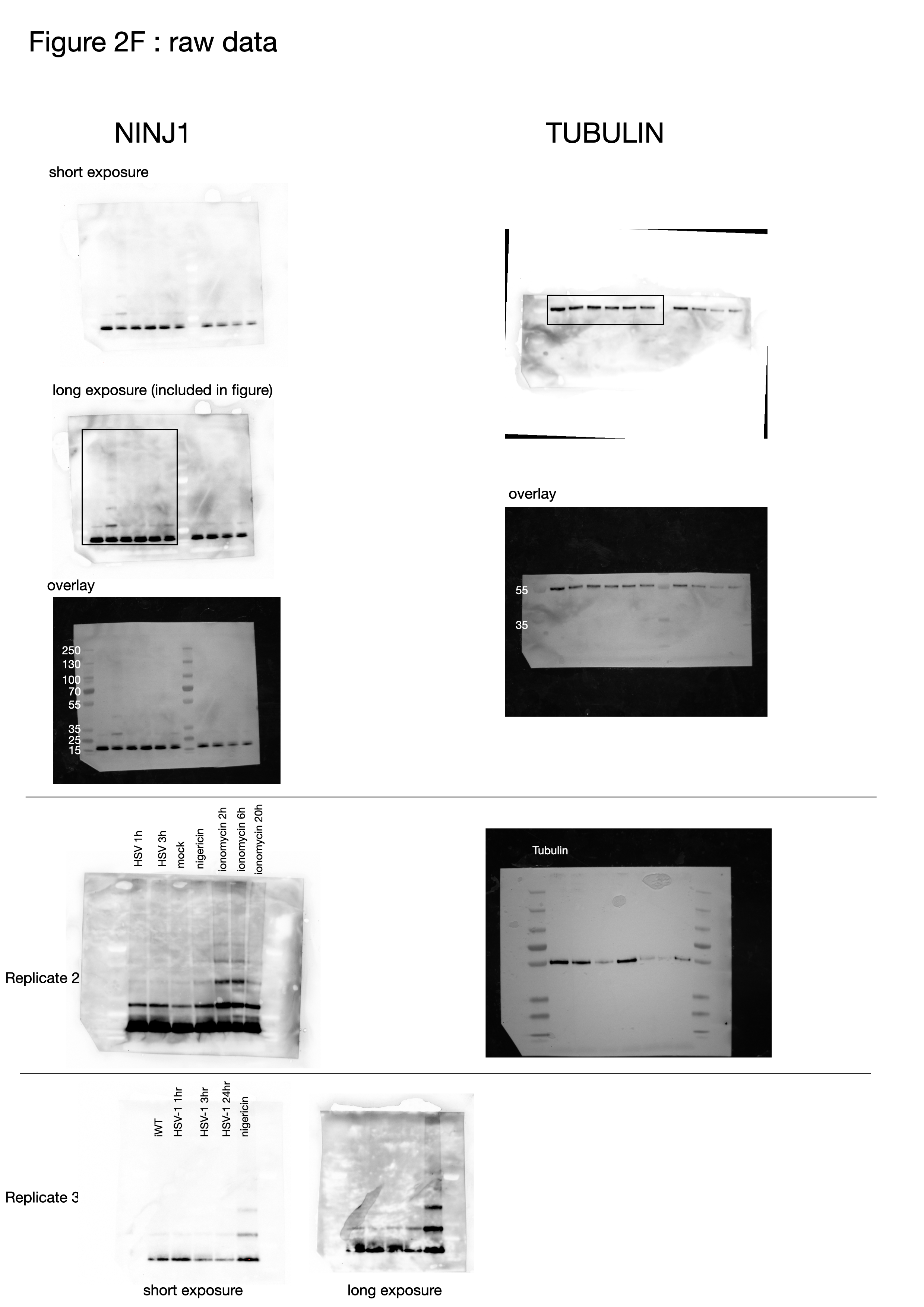

Supplement: Supplementary file 3 — Source data Fig. 2 [file 44319_2025_638_MOESM3_ESM.zip › Figure 2/Fig2F.tiff]

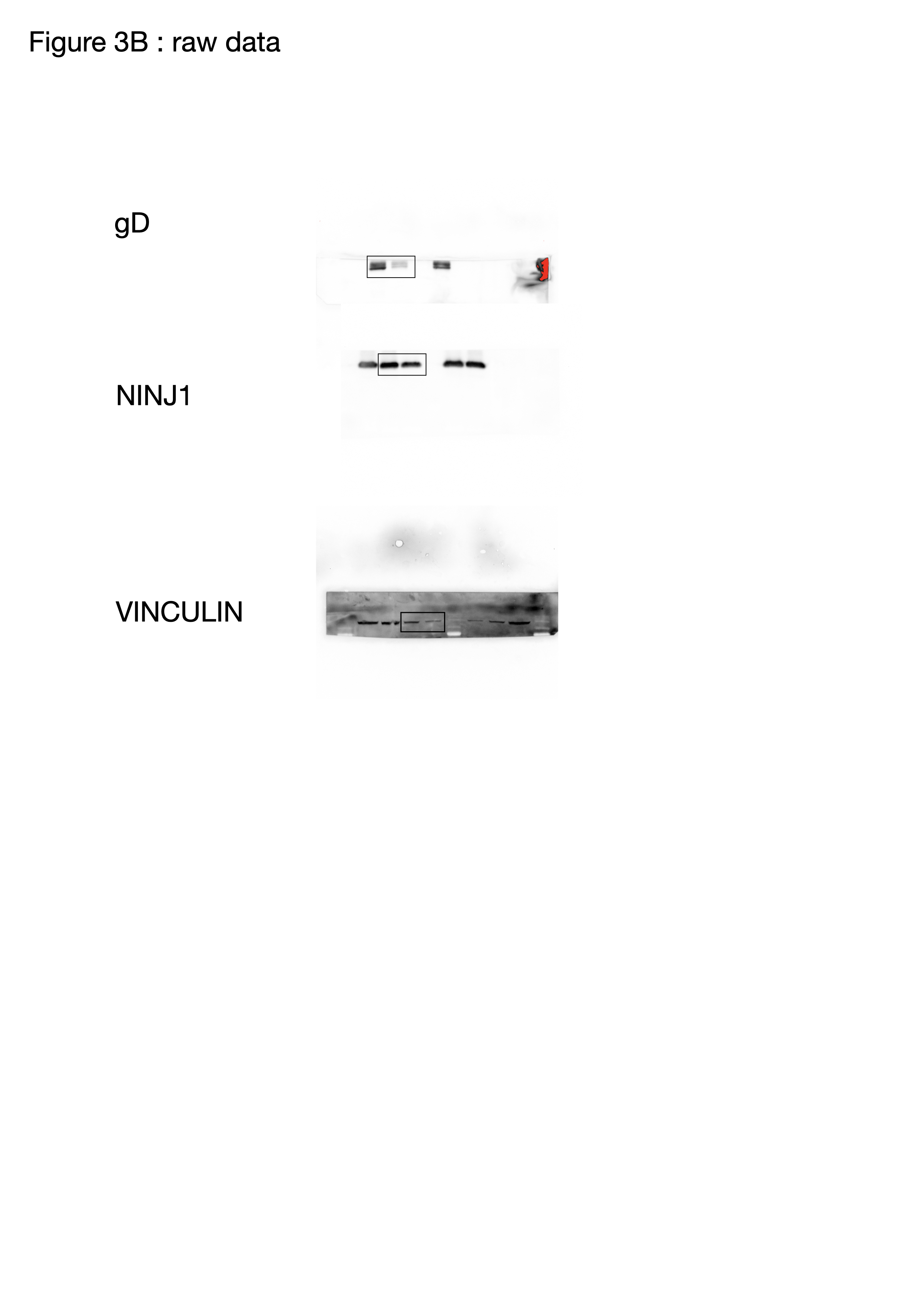

Supplement: Supplementary file 4 — Source data Fig. 3 [file 44319_2025_638_MOESM4_ESM.zip › Figure 3/Fig3B.tiff]

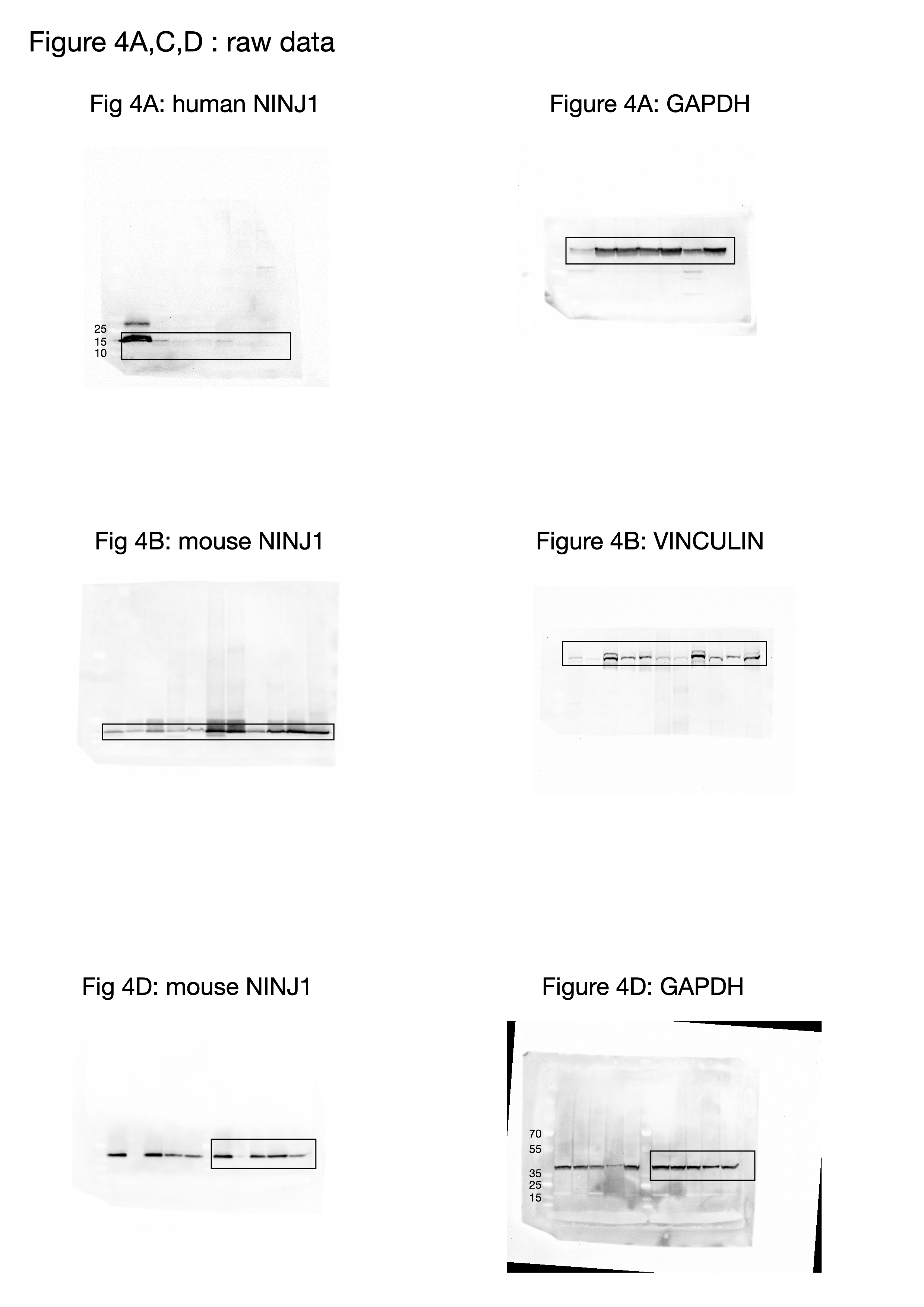

Supplement: Supplementary file 5 — Source data Fig. 4 [file 44319_2025_638_MOESM5_ESM.zip › Figure 4/Fig4A,C,D.tiff]
