# Supplementary material for: NINJ1 blocks HSV-1 entry into macrophages to impact viral replication and immunity
Source: EMBO Rep. 2025 Nov 19;27(1):69–88. doi: 10.1038/s44319-025-00638-8 (PMC12796307; doi:10.1038/s44319-025-00638-8)
Supplement: Supplementary file 7 — Expanded View Figures [file 44319_2025_638_MOESM7_ESM.pdf]

Expanded View Figures

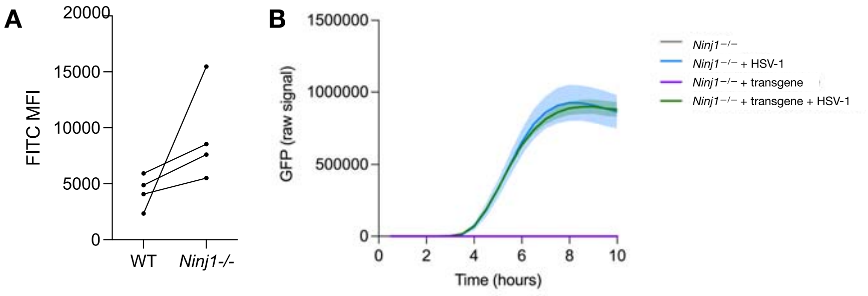

Figure EV1. NINJ1 controls the susceptibility of mouse macrophages to HSV-1.

(**A**) WT and *Ninj1*$^{-/-}$ iBMDMs were infected for 6 h with HSV-1 GFP, subjected to flow cytometry and the median fluorescence intensity was measured in FlowJo. (**B**) *Ninj1*$^{-/-}$ iBMDMs ± integration of a blasticidin resistance transgene were infected with HSV-1 GFP, and GFP signal was measured every 10 min on a Citation5 Plate Reader. Data information: (**A**) dots are biological replicates with a line connecting data collected as part of the same experiment, (**B**) line is mean ± SD from three technical replicates, representative of two biological replicates.

 © The Author(s)

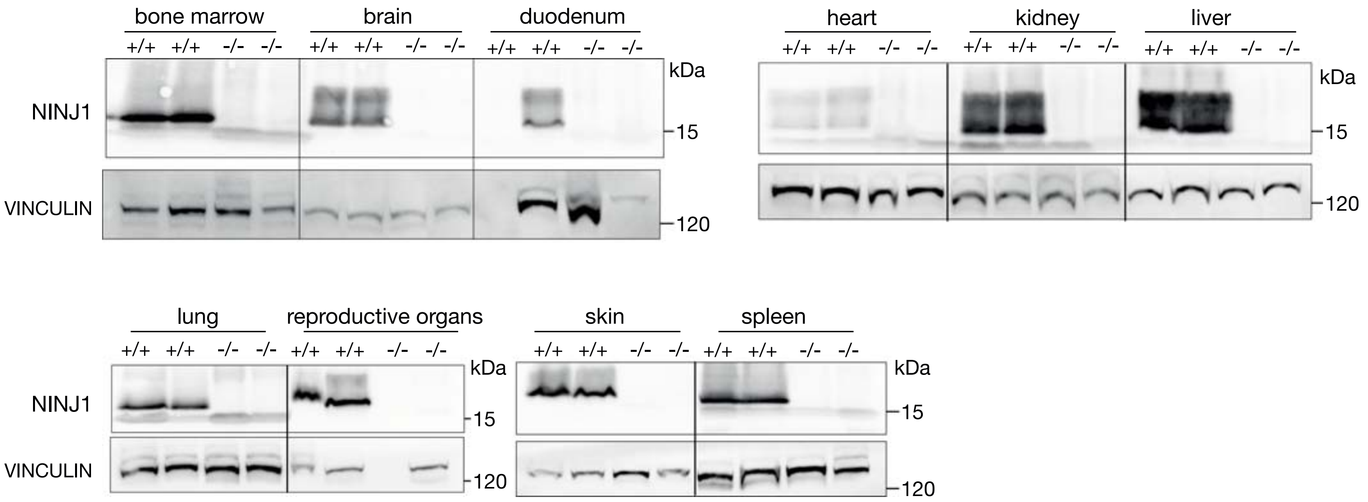

Figure EV2. NINJ1 is highly expressed in primary macrophages and mouse tissue.

Organ homogenates from two WT mice (+/+) and two *Ninj1*$^{-/-}$ mice (−/−), one male (left) and one female (right) of each genotype, were subjected to immunoblot for NINJ1 expression across the indicated tissues.

© The Author(s)

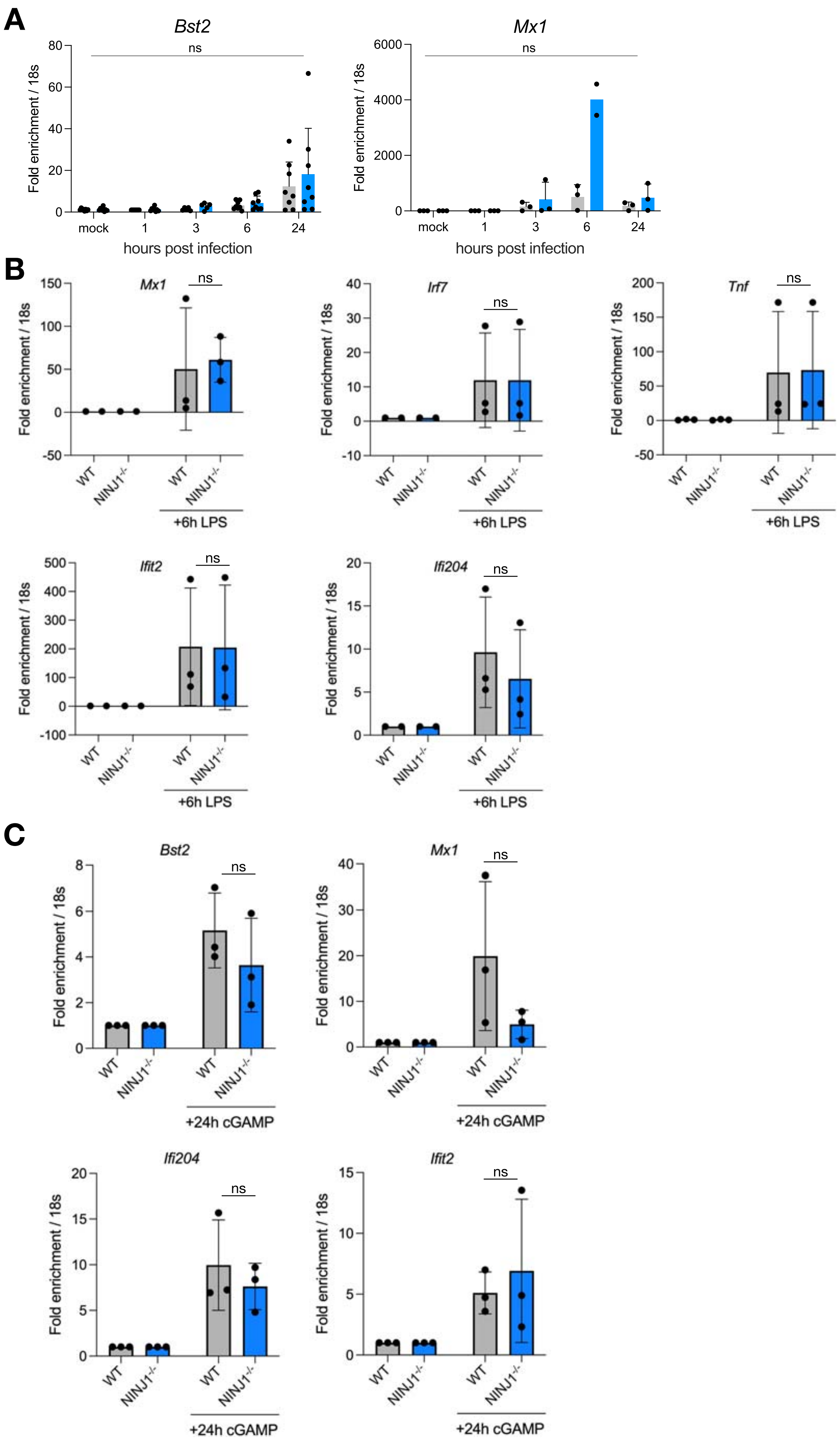
A
Bst2
Mx1
ns
Fold enrichment / 18s
hours post infection
mock
1
3
6
24
B
Mx1
Irf7
Tnf
Ifit2
Ifi204
WT
NINJ1-/-
+6h LPS
C
Bst2
Mx1
Ifi204
Ifit2
+24h cGAMP

 © The Author(s)

Figure EV3. ISG transcript levels are higher in *Ninj1*$^{-/-}$ *cells* upon HSV-1 infection.

(**A**) WT and *Ninj1*$^{-/-}$ iBMDMs were infected for the indicated times with HSV-1 GFP, and RNA levels of the indicated transcripts were measured by RT-qPCR. (**B**) WT and *Ninj1*$^{-/-}$ iBMDMs ± stimulation with 10 µg/mL LPS for 6 h. RNA was harvested and subjected to qPCR for the indicated transcripts. (**C**) WT and *Ninj1*$^{-/-}$ iBMDMs ± stimulation with 5 µg/mL 2′3′cGAMP for 24 h. RNA was harvested and subjected to qPCR for the indicated transcripts. Data information: (**A**–**C**) mean ± SD is plotted with dots showing biological replicates, paired *t*-tests, ns not significant. (**A**) $n = 3$–8, (**B**, **C**) $n = 3$.

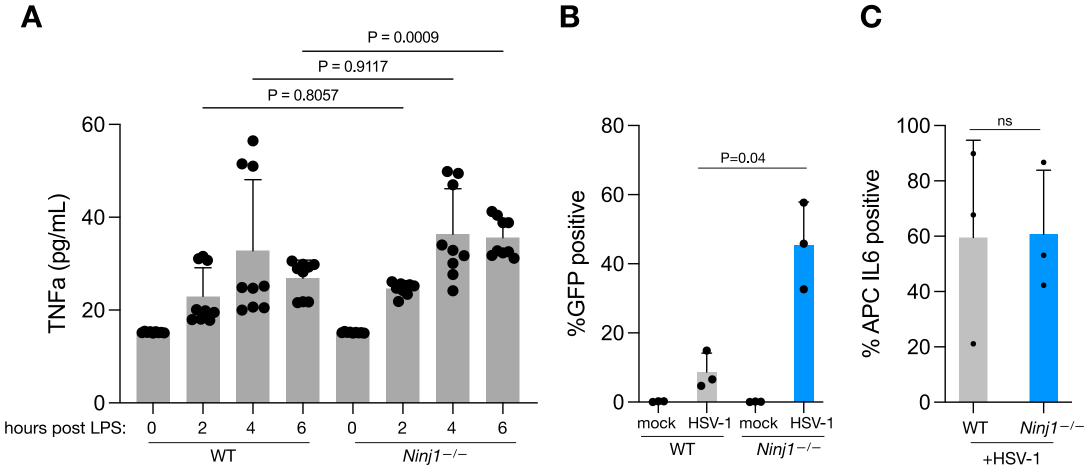

Figure EV4. Loss of NINJ1 results in lower cytokine secretion.

(**A**) WT and *Ninj1*$^{-/-}$ iBMDMs were stimulated with 10 μg/mL LPS for the indicated periods of time. Supernatant were harvested and subjected to an ELISA for TNFα. Bars show the mean and SD. (**B**, **C**) WT and *Ninj1*$^{-/-}$ iBMDMs were infected for 6 h and then stained for intracellular IL-6. Cells were analyzed on a flow cytometer for GFP levels (**B**) and APC IL-6 levels (**C**). Data information: (**A**–**C**) mean ± SD is plotted. A dots show three technical replicates from three biological replicates, Brown-Forsythe and Welch ANOVA. (**B**, **C**) dots show biological replicates, unpaired *t*-tests, ns not significant, $n = 3$.

 © The Author(s)
